# Hybrid Macrocyclic Peptides — Synthetic Strategies to Diversify and Cyclize Peptide Libraries in Phage Display Selections

**DOI:** 10.1002/chem.71408

**Published:** 2026-07-14

**Authors:** Titia Rixt Oppewal, Clemens Mayer

**Affiliations:** ^1^ Stratingh Institute for Chemistry University of Groningen Groningen The Netherlands

**Keywords:** bio‐orthogonal chemistry, macrocyclic peptides, macrocyclization, phage display, protein modification

## Abstract

Macrocyclic peptides (MPs) are privileged molecular architectures in drug discovery that combine the target selectivity of biologics with the pharmacological advantages of small molecules. However, peptide binders identified by biological selection platforms such as bacteriophage display often require extensive post‐selection medicinal chemistry optimization before entering the clinic. In particular, the incorporation of nonpeptidic scaffolds and synthetic cyclization units is frequently necessary to improve binding affinity, proteolytic stability, membrane permeability, and overall pharmacological performance. In this Review, we provide a comprehensive overview and critical analysis of strategies that enable the direct selection of such *hybrid* MPs by bacteriophage display. We discuss established cysteine‐ and lysine‐targeting chemistries, recent advances in residue‐selective and chemoenzymatic cyclization methods, and emerging approaches that exploit proximity‐driven reactivity or the chemical sturdiness of bacteriophages. Collectively, these strategies enable the simultaneous exploration of peptide sequence and synthetic chemical space during biological selections, thereby potentially reducing downstream optimization efforts. Finally, we outline key design principles for phage‐compatible macrocyclization reactions and discuss future opportunities at the interface of bioconjugation chemistry, enzyme engineering, and machine‐learning‐guided peptide discovery to accelerate the development of next‐generation peptide therapeutics.

## Graphical abstract

Hybrid macrocyclic peptides unite genetically encoded peptide libraries with the structural complexity of synthetic small molecules. This Review critically analyzes phage‐compatible cyclization and diversification chemistries, highlights emerging opportunities beyond traditional cysteine‐based approaches, and outlines how future advances may enable the discovery of peptide therapeutics with tailored pharmacological properties.

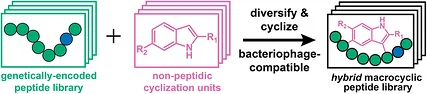

## 1 Introduction

### 1.1 The Three Waves of Therapeutic Peptide Discovery

Since the isolation of insulin over a century ago, the field of peptide therapeutics has significantly advanced (Figure 1A) [1, 2, 3, 4]. Early efforts were largely restricted to evaluating naturally occurring peptides or their close analogues, due to challenges in *de novo* peptide synthesis. As such, peptides were initially applied as therapeutics in areas where natural peptides predominate, such as metabolic regulation, immunological control, or to combat infectious diseases [2].

**FIGURE 1:**
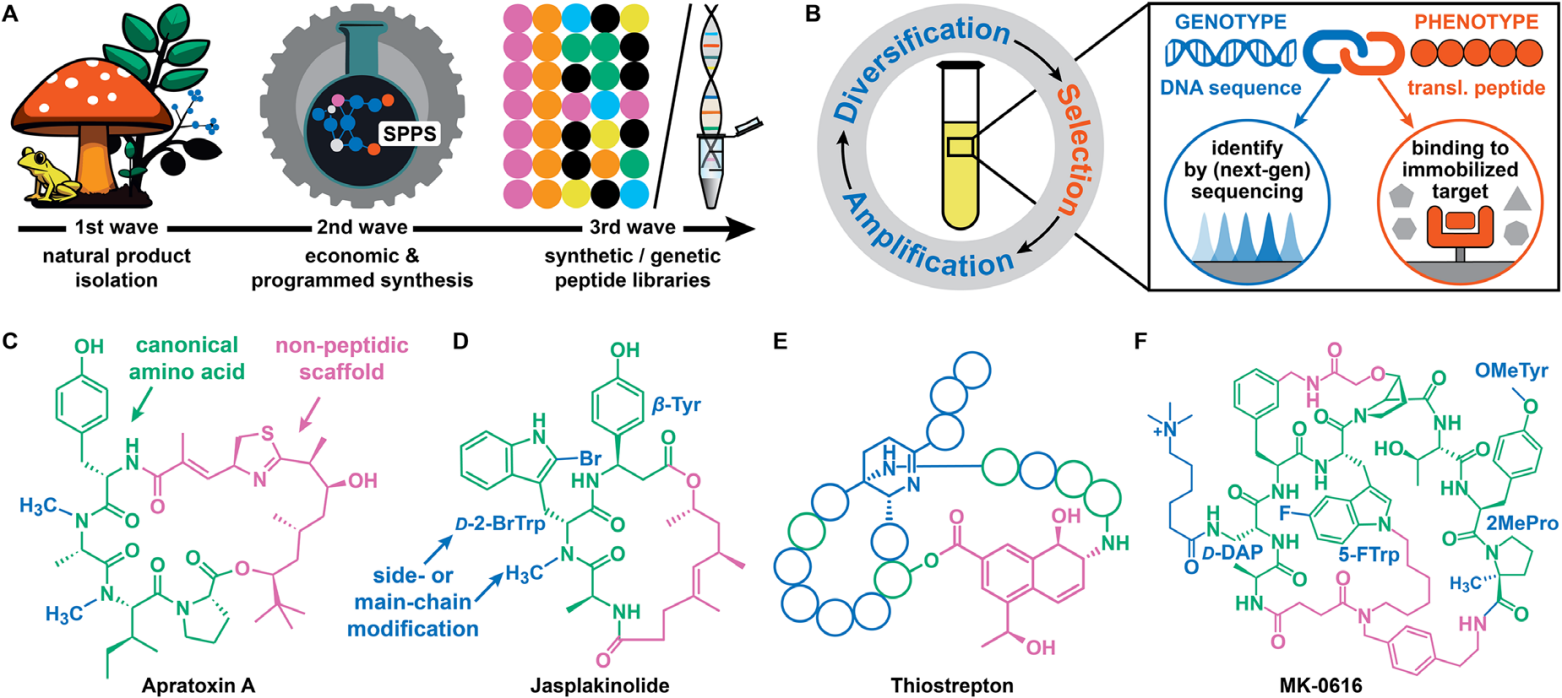
History, principles, and examples of therapeutical (macrocyclic) peptides. (A): The three waves of peptide pharmaceuticals differ in the isolation, synthesis, and discovery of peptide binders against disease‐related targets. (B): Biological selections recapitulate the principal steps of the evolutionary algorithm in a test tube. A genotype‐phenotype link is central to evaluate the binding properties of vast peptide libraries in parallel and identify binding peptides based on their DNA sequences. (C‐F): Structures or representations of natural and man‐made MPs that in addition to canonical amino acids (green), feature side‐ or main‐chain modifications (blue) as well as nonpeptidic cyclization units (pink).

The field underwent substantial growth through the development of solid‐phase peptide synthesis (SPPS), a technique in which activated amino acids are coupled one‐by‐one to a growing peptide chain that is immobilized on a solid support (Figure 1A) [5]. SPPS not only enabled the on‐demand synthesis of diverse peptides, but also substantially lowered the costs for their large‐scale production [6]. Their straightforward synthesis also facilitated the rational design of peptide therapeutics by accessing, modifying, and evaluating fragments/motifs of known protein‐binders [1, 2].

The production and evaluation of large peptide libraries represented the *third wave of peptide therapeutics* and enabled the *de novo* discovery of peptide binders against targets of interest (Figure 1A) [7]. For example, by employing chemical strategies, such as one‐bead‐one‐compound (OBOC) [8] approaches or DNA‐encoded libraries (DELs) [9], peptide libraries can be synthesized and subsequently evaluated for their ability to bind to a target of interest. Conversely, as peptides are genetically encodable, vast libraries can also be efficiently produced and culled by biological selections, such as various *display* strategies or split‐intein circular ligation of peptides and proteins (SICLOPPS) [10].

Together, these innovations have driven the rapid growth of peptide therapeutics, contributing to a flourishing global market that is expected to grow from $141 billion in 2025 to ∼$295 billion in 2033 [11]. This continuous success is the result of peptides occupying a chemical space between biologics and small‐molecule drugs, and having the potential to combine the beneficial properties of both [4, 12]. Specifically, therapeutic peptides, like antibodies, can bind targets with high selectivity and specificity, yet they are smaller in size and often cheaper to manufacture. Additionally, difficult targets such as protein‐protein interfaces — generally considered *undruggable* for small‐molecule drugs — can be targeted by peptide therapeutics. Lastly, peptides degrade into amino acids, which minimizes the risk of adverse side effects and build‐up of toxic metabolites.

### 1.2 Directed Evolution—How Scientists Became Custodians of Diverse Populations of Evolving Chemicals

Biological selections have proven a powerful tool to elicit potent peptide binders against a wide array of therapeutically‐relevant targets. Such *directed evolution* approaches generate made‐to‐order biomolecules by recapitulating the three steps of the Darwinian algorithm: diversification, selection, and amplification of successful variants (Figure 1B) [13]. Rapid advances in molecular biology operations over the past few decades have rendered the *de novo* selection of peptide binders feasible, realizing the vision of George P. Smith, who imagined *the applied chemist, not as designer of molecules with a particular purpose, but rather as custodian of a highly diverse population of chemicals evolving* in vitro *as if they were organisms subject to natural selection* [14].

Indeed, compared to the laborious process of designing, synthesizing, and evaluating peptides one‐by‐one, biological selection platforms enable the straightforward production of vast genetically encoded peptide libraries containing 10^8–^10^13^ unique members [7, 10]. A robust link between genotype (DNA sequence) and phenotype (translated peptide) is crucial to ensure the genetic ‘barcoding’ of individual peptide variants within these vast libraries (Figure 1B). Then, akin to how impotent natural compounds are weeded out from the chemical pool by natural selection, tight binders can be identified from genetically‐encoded peptide libraries by incubating them with immobilized (drug) targets of interest. After nonbinders are removed by affinity selection, the identity of binding peptides can be determined by (next‐generation) sequencing of their corresponding genetic barcode (Figure 1B).

### 1.3 Learning From Nature for Hit‐To‐Lead Optimization Strategies

While biological selections have rendered the identification of peptides that bind a target with high affinity routine, their elaboration into pharmaceuticals typically presents a number of obstacles [15, 16, 17]. First, peptides are polar and display poor pharmacological properties, such as limited membrane permeability and oral bioavailability. In addition, peptides often possess a short circulating half‐life due to proteolytic degradation as well as rapid renal and hepatic clearance. To address these and other challenges, medicinal chemists often take inspiration from nature, where peptides frequently undergo elaborate (posttranslational) modifications to improve their pharmacological properties.

For example, macrocyclization is a ubiquitous strategy that often enhances the bioactivity of peptide natural products. (Figure 1C‐E) [15, 18]. In fact, macrocyclic peptides (MPs) not only show higher binding affinities than their linear counterparts, but also exhibit significantly improved stability and membrane permeability. To further increase bioavailability, backbone amides are often sequestered either by (1) hydrogen bond donors and acceptors masking each other, a phenomenon known as chameleonic behavior [19], (2) the incorporation of hydroxy‐acids to form depsipeptides [20], or (3) by N‐methylation (e.g. Apratoxin A, Figure 1C) [21], To aid MPs to fold into distinct conformations and prevent recognition by endogenous proteases, noncanonical *d*‐ or β‐amino acids feature prominently in naturally‐occurring MPs (e.g. Jasplakinolide, Figure 1D). Lastly, many natural MPs found incorporate nonpeptidic scaffolds (e.g. secondary metabolites) that can serve as cyclization units, contribute unique binding interactions, and improve overall pharmacokinetic properties (e.g. Figure 1C‐E).

Over the past decades, these strategies have been successfully applied by medicinal chemists to optimize peptide leads obtained from display selections [3, 22] A notable recent example of such a medicinal chemistry campaign resulted in MK‐0616 (Figure 1F), an orally‐available PCSK9 inhibitor developed by *Merck & Co* [23]. Based on an MP lead compound identified by mRNA display (*K*_i_ = 956 nM), initial optimization steps relied on structure‐activity‐relationship studies, which pinpointed residues that could either be removed or replaced with (non)canonical amino acids to increase affinity and stability (*K*_i_ = 4.2 nM) [24]. However, the biggest potency gains resulted from restricting conformational freedom further through two additional cyclizations (*K*_i_ = 5 pM) [25, 26]. Notably, these additional cyclizations were achieved using nonpeptidic scaffolds that were critical for tuning the pharmacokinetic properties of MK‐0616 (Figure 1F).

### 1.4 Definitions, Scope, and Aims of this Review

The example of MK‐0616 demonstrates that lead optimization campaigns performed by medicinal chemists result in structures that combine a (ribosomally‐derived) peptide core with nonpeptidic chemical moieties, such as synthetic cyclization units or secondary metabolite‐like scaffolds. Such *hybrid* MPs (Figure 2) mimic bioactive compounds found in nature, for which eons of evolution have evolved biosynthetic gene clusters capable of diversifying and cyclizing linear peptides through the incorporation of nonpeptidic building blocks. Given the similarities between natural and man‐made compounds (Figure 1C‐F), we surmise that such hybrid MPs occupy a privileged chemical space that is rich in structural diversity, target engagement potential, and pharmacokinetic versatility. Critically though, searching this chemical space at scale has proven challenging, as the modification and cyclization of genetically‐encoded peptide libraries with diverse, nonpeptidic scaffolds is not straightforward.

**FIGURE 2:**
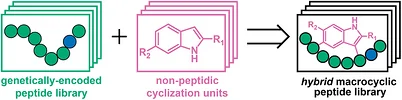
Hybrid MPs. For this review, we consider hybrid MPs entities that result from the cyclization of (non)natural, linear peptides with nonpeptidic cyclization units.

In this review, we aim to provide an in‐depth analysis of existing and yet un(der)explored approaches for the selection of natural‐product‐like, hybrid MPs (Figure 2). While in principle applicable to all biological selection strategies, we focus on phage display for which synthetic cyclization strategies have thus far proven most versatile. Following a concise overview of relevant theoretical and practical aspects of phage display for the uninitiated reader, we highlight established synthetic methods for diversifying and cyclizing MPs with nonpeptidic scaffolds directly on phage particles. Subsequently, we survey the literature for promising cyclization strategies that could be applicable in biological selections. Lastly, we outline key design criteria that cyclization strategies should meet to enable the discovery of bioactive, hybrid MPs and, based on this analysis, chart the course toward a possible fourth wave of peptide therapeutics.

## 2 Phage Display

All selection strategies that are able to elicit peptide binders from large libraries rely on (parts of) nature's protein production machinery and a link between the encoding DNA and the translated peptide (Figure 1B) [7, 10]. In bacteriophage display, this genotype‐phenotype link is achieved by genetically fusing peptides or proteins of interest to a phage coat protein, with their respective DNA sequences being encoded and protected in the phage genome [14]. While by themselves unable to re‐produce, phages can readily infect bacteria, such as *Escherichia coli*, and take advantage of the host's translation machinery to provide a multitude of progeny from a single infection event. The following sections discuss how these characteristics have been exploited to make phage display a reliable strategy for the selection of peptide binders and compares the use of bacteriophages as genotype‐phenotype link to other display technologies.

### 2.1 Theoretical and Practical Aspects

*For the Phage Display of Peptides and Antibodies* George P. Smith and Gregoy P. Winter were jointly awarded half of the 2018 Nobel Prize in Chemistry [14, 27, 28]. Their pioneering efforts paved the way to covalently link vast peptide libraries with up to 10^9^ unique variants to the surface of filamentous bacteriophages, which are rod‐shaped, nonlytic viruses that infect bacteria (Figure 3A) [29]. In practice, this display is achieved by appending a peptide‐encoding, randomized oligonucleotide sequence to the 5’ (for N‐terminal display) or 3’‐ end (for C‐terminal display) of a gene encoding for one of the phage coat proteins [30].

**FIGURE 3:**
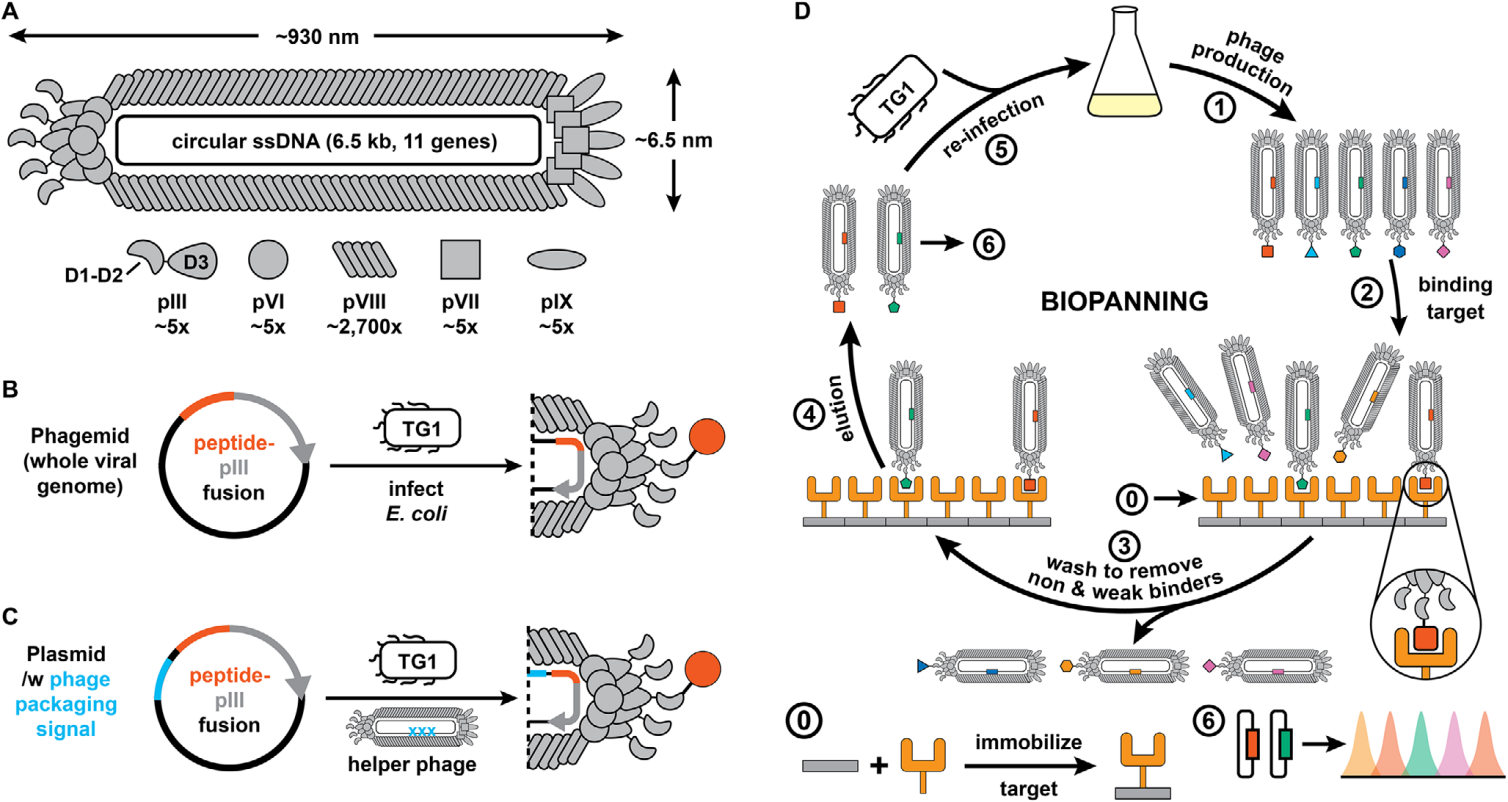
Principles of bacteriophage display. (A): Schematic representation of filamentous bacteriophages displaying coat proteins involved in protecting the viral genome. (B): Genetic fusion between a peptide and the pIII protein results in the production of phages that display linear peptides on their coat. (C): Combining a plasmid with a phage packaging signal and a helper phage can produce replication impaired bacteriophages that display a lower number of linear peptides. This strategy is used for displaying large N‐terminal proteins and to avoid the formation of replicative species in bacteriophage display. C: Overview of a phage display cycle. Following phage production (step 1), biopanning (steps 2–4) gives rise to a phage population enriched in binding peptides, which can be used to re‐infect bacteria (step 5). Immobilization of the target protein (step 0) and identification of enriched peptides by sequencing (step 6) are outside the phage display cycle.

Filamentous bacteriophage, such as M13 or fd, possess multiple coat proteins that have been explored as fusion partners for peptide libraries (Figure 3A) [31]. Among these, the minor coat protein pIII has been most successfully employed in phage display selections. With 3–5 copies per virion, appending peptide libraries to pIII elicits binders with high affinities and also reduces avidity effects, where multiple copies of a peptide simultaneously contribute to binding. In contrast, display strategies utilizing pVIII, which appears ∼2,700 times per assembled phage particle, often yield lower‐affinity or nonspecific binders due to high multivalency. Additionally, N‐terminal insertions into pIII are typically well tolerated, with the small genome of filamentous phages (around 6,400 nucleotides) also lending itself to routine molecular biology operations. Lastly, M13 and fd are nonlytic phages that replicate in *E. coli* without killing the host, which enables the accumulation of a high number of replicative phages (i.e. phage titer) [31, 32].

Multiple strategies have been developed over the past decades to achieve N‐terminal display of peptide libraries on pIII. In the original approach, genes encoding for the peptide‐pIII‐fusion and all other structural/regulatory proteins are placed on a single plasmid, known as phagemid (Figure 3B) [14]. While effective, displaying larger sequences can interfere with proper assembly of phage particles, due to steric demands and/or lower expression levels of the fusions relative to other phage structural proteins. To overcome these limitations, a two‐plasmid system can be employed, which makes use of a plasmid that encodes for the pIII‐fusion and a *helper phage*, which contains all structural and functional phage genes (Figure 3B) [31, 33]. As pIII is produced from both components, this setup can be employed to artificially lower the number of pIII‐peptide fusions presented on an intact virion. Additionally, the plasmid, but not the helper phage harbors the packaging signal, ensuring that only the former is packed into the produced phage particles. As such, this strategy prevents that selections give rise to replicative species, as produced virions are infective but cannot generate progeny in the absence of the helper phage.

Independent of the approach used, *E. coli* transformed with appropriate plasmids will produce phage particles that display the peptides, which correspond to the randomized nucleotide sequence appended to the pIII gene in their packaged plasmid. The resulting phage libraries are subsequently subjected to consecutive rounds of an affinity selection regime called *biopanning* (Figure 3C). In this process, phage libraries are incubated with a surface‐immobilized target of interest, which allows peptide sequences with affinity for this target to bind. Next, phages displaying non or weakly binding peptides are removed by repeated washing steps with buffers that typically contain detergents. Virions displaying binding peptides are then eluted, for example, by lowering the pH to ∼2. Finally, the recovered phages are used to reinfect new host cells, enabling the specific amplification of binding over nonbinding peptides over one phage‐display cycle. This enriched population can be subjected to further rounds of biopanning (3‐5 in total) to elicit the binders with the highest affinity from the original population.

(Next‐generation) Sequencing of enriched pIII genes from phage display campaigns often reveals peptide binders that feature identical or similar amino acid sequences, which are critical for binding [34]. Conversely, regions of peptides, which show less convergence can subsequently be targeted for randomization in a follow‐up phage display campaign. Such an *affinity maturation* of initial hits typically elicits binders with improved affinities, particularly if the theoretical size of the initial peptide library exceeded the number of variants that can be covered by phage display at once (10^9^ variants corresponding to 6 randomized amino acids). Overall, phage display has proven a fast and robust means to generate *de novo* binders for targets of interest. A full cycle, going from library preparation to deep sequencing, typically takes about one month.

### 2.2 Advantages and Disadvantages to Other Display Strategies

The development of phage display has spurred researchers to explore other genotype‐phenotype links that are suitable for the *de novo* identification of desired phenotypes. For example, peptides and proteins have successfully been displayed on the outside of bacteria [35], yeast [36], and mammalian cells [37]. These approaches can be advantageous when appending proteins that are large and/or otherwise difficult to produce (e.g. antibodies), but have been less frequently employed in the selection of peptide binders [38]. Other strategies bypass the need to transform replicating host cells altogether by performing all steps of the Darwinian algorithm in vitro [39]. Prominent examples include ribosome and mRNA display, of which the latter has proven particularly powerful for the selection of linear and cyclic peptides [40].

Developed by Roberts and Szostak, mRNA display takes advantage of the antibiotic puromycin to generate a covalent link between an mRNA and its encoded peptide [41]. In brief, puromycin mimics an aminoacylated tRNA and enters the ribosome's active site, where it accepts the growing peptide chain, prematurely terminating translation. To repurpose puromycin for the generation of a genotype‐phenotype link, mRNA display makes use of hybrid constructs that consist of (1) a peptide‐encoding mRNA, (2) a stuffer DNA fragment, and (3) a 3’ puromycin modification [42, 43]. When subjecting the construct to conditions for in vitro translation, the ribosome stalls at the mRNA/DNA junction, enabling puromycin to enter the ribosome's active site and become covalently attached to the growing peptide chain. This mode‐of‐action covalently links the translated peptide to the mRNA/DNA construct, enabling identification of binding peptides by affinity selection against an immobilized target. Reverse‐transcription followed by (error‐prone) PCR amplification enables the (diversification and) identification of binding sequences. mRNA display has evolved substantially since its introduction [44]. Most notably, efforts led by the Suga lab have led to development of the Random nonstandard Peptide Integrated Discovery (RaPID) platform [45], which has enabled the generation of vast, MP libraries containing a wide variety of exotic and privileged noncanonical building blocks [46, 47, 48].

While the RaPID system offers unparalleled library sizes (up to 10^1^ ^2^ variants) and can incorporate a wide variety of amino‐ or hydroxy‐acids as well as peptidomimetics into MP discovery campaigns, it requires significant technical expertise. In contrast, phage display is operationally accessible and can be established in nonspecialist laboratories. In addition, the RaPID workflow depends on sensitive and expensive reagents, while phage display relies on cheaper components and virions readily withstand elevated temperatures, treatment with oxidative/reductive reagents, mildly basic/acidic pHs, and the presence of up to 50% co‐solvents [49, 50]. This chemical robustness is a key reason why phage display has become a powerful platform for the selection of hybrid MPs, as it permits on‐phage chemical modification and cyclization without compromising virion integrity.

## 3 Phage‐Compatible Cyclization Strategies Employing Symmetrical Cross‐Linkers

### 3.1 Balancing the Reactivity and Selectivity of Reagents for Peptide Macrocyclization

Conceptually, accessing hybrid MPs requires the use of nonpeptidic scaffolds that can react with side‐chains and/or termini of linear precursors displayed on phages. To do so, potential cyclization units are equipped with functional groups that must strike a fine balance between their reactivity and selectivity. In terms of reactivity, cyclization reactions on phage particles should proceed under dilute conditions, with typical phage titers of 10^11–^ 12 phage particles per mL corresponding to a peptide concentration of ∼0.1‐1 nm. With respect to selectivity, nonpeptidic scaffolds must react with only two residues in a virion composed of thousands of proteins and a molecular weight of ∼16 MDa [14]. As such, adding reactive cyclization units in large excess can drive reactions to completion but also increases the risk of nonspecific modifications. Conversely, using lower concentrations improves selectivity but exponentially increases reaction times. Moreover, phage infectivity cannot be jeopardized by the scaffold, additives, or reaction conditions, as the amplification of binding MPs following biopanning is crucial for the overall success of phage display.

Taken together, achieving fast and selective cyclization with a nonpeptidic scaffold is essential, yet represents a formidable challenge. Not surprisingly, many successful phage‐compatible cyclization strategies draw inspiration from site‐selective protein bioconjugation methods, which face similarly stringent requirements for chemoselectivity under complex, dilute, and biologically relevant conditions [51, 52, 53]. We begin by examining strategies that use symmetrical cross‐linkers, an approach that offers synthetic simplicity and favorable reaction kinetics under phage‐compatible conditions.

### 3.2 Cysteine Alkylation—A Workhorse for Phage‐Compatible Macrocyclization

Cysteines are a mainstay in bioconjugation reactions due their uniquely nucleophilic thiol side chains [54]. Indeed, cysteines undergo fast and selective alkylation at near neutral pH in presence of soft electrophiles. Among these, conjugate additions with α/β‐unsaturated carbonyls or nucleophilic substitution with aryl‐ or alkyl‐halides are most commonly employed. Moreover, cysteines have low abundance across proteins (<2%), thus minimizing side reactions when cyclizing linear precursors on phage particles [55]. For synthetic peptides (and proteins), cyclization reactions typically make use of symmetrical bis‐electrophiles as reactive scaffolds, which can crosslink or *staple* two nearby cysteines (Figure 4A) [56]. However, as adjacent thiols readily oxidize to disulfides, a reducing agent is typically required to regenerate free thiols prior to cyclization.

**FIGURE 4:**
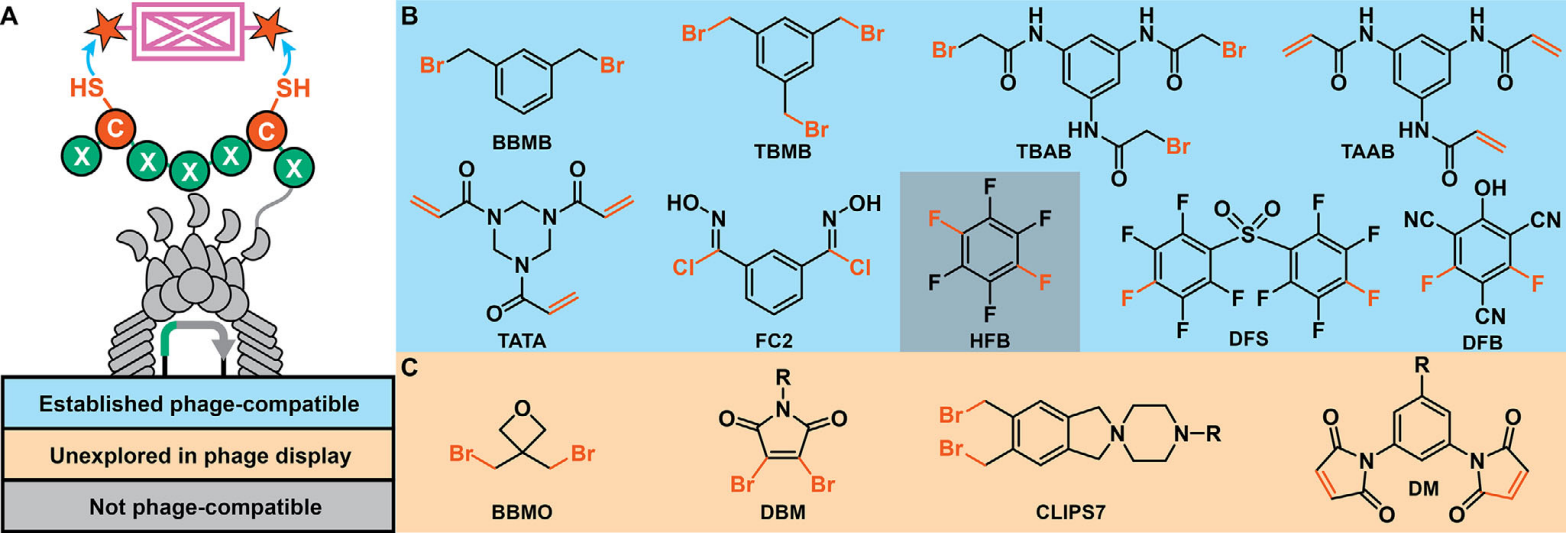
Phage‐compatible cyclization employing cysteine crosslinkers. (A): Schematic representation of on‐phage cyclization employing symmetrical cysteine crosslinkers (B): Structures of cyclization units evaluated or employed in phage display campaigns to select hybrid MP binders. (C): Structures of symmetrical cysteine crosslinkers that have thus far remained unexplored in the selection of hybrid MPs.

Unfortunately, the required reducing agents, such as tris(2‐carboxyethyl)‐phosphine (TCEP), severely impact phage infectivity and have historically limited the use of disulfide‐reducing conditions for cyclizing cysteine‐containing peptides directly on phages. This susceptibility to TCEP can largely be attributed to the reduction of three stabilizing disulfide bonds in the D1‐D2 domains of pIII, which are essential for the initial steps of the phage infection cycle [57]. Additionally, the D1‐D2 domains are also the site of fusion for peptide libraries, with displayed sequences featuring unpaired cysteine residues having been shown to disrupt folding of pIII [58]. To overcome these challenges, Kather, Bippes, and Schmid performed a directed evolution campaign to engineer pIII proteins featuring stable, yet disulfide‐free D1D2 domains [59]. While titers of these engineered phages were markedly reduced (0.1%–1% of wild type filamentous phages) and infection cycles notably slower (90 min. instead of 10 min. for wild type), these parameters were largely unaffected by the addition of reducing agents [60].

Recognizing the potential of such *disulfide‐free* phages, Heinis and coworkers displayed cysteine‐containing peptides on their surface. After reduction with TCEP, they explored cyclization reactions with symmetric crosslinkers, such as 1,3‐bis(bromomethyl)benzene (**BBMB**) and 1,3,5‐tris(bromomethyl)benzene (**TBMB,** Figure 4B) [61]. In these efforts, they were guided by the works of Kemp and McNamara, who employed **TBMB** to furnish bicyclic nonapeptides from (Gly‐Cys‐Gly)_3_ [62], and Timmerman and coworkers, who demonstrated the excellent selectivity of electrophilic crosslinkers for cysteine residues in aqueous conditions [63]. Ultimately, Heinis and coworkers performed their (bi)cyclization reactions on phages in aqueous buffer at pH 8 (20% ACN) for 1 h at 30°C. Encouragingly, phage infectivity was only reduced five‐fold at a TBMB concentration of 10 µm, with higher concentrations drastically decreasing measured phage titers. This negative impact on the phage lifecycle is suspected to be the result of TBMB also crosslinking solvent‐exposed lysine residues on phage particles.

Excitingly, when performing cyclization reactions with a randomized peptide library featuring three cysteine residues, biopanning cycles elicited potent inhibitors for the protease human plasma kallikrein (*K*_i_ = 1.5 nm). When synthesized in vitro, the resulting bicyclic constructs displayed high stability and, consistent with a reduced entropic penalty for binding/folding, proved to be around 250‐fold more potent than their linear counterparts [61].

Following this initial proof‐of‐concept, Chen and coworkers successfully employed threefold‐symmetrical crosslinkers other than **TBMB** in the selection of bicyclic peptides [64, 65]. In addition to cyclization units that undergo nucleophilic substitution (**TBAB**), they also applied tri‐symmetrical linkers featuring α/β‐unsaturated carbonyl moieties (**TATA** and **TAAB**, Figure 4B). While compounds that undergo Michael addition displayed high selectivity for cysteines, the resulting bicyclic constructs were found to be less stable under biologically‐relevant conditions [66, 67]. Additionally, the oxidation of the thioethers introduced by either strategy presents a potential obstacle, particularly when upscaling the production of bicyclic lead compounds [68].

Since these pioneering reports, a number of groups have explored alternative scaffolds that can be used to diversify and cyclize linear peptide libraries displayed on phage particles (see Figure 5). For example, Chen and colleagues reported a fast and selective cysteine crosslinking strategy making use of chlorooximes (**FC2**, Figure 4B) [69, 70]. Specifically, they were able to efficiently cyclize peptides displayed on whole bacteriophages in 30 min using only 10 µm of their lynchpin molecule in PBS buffer at pH 7.4. While the chlorooxime scaffold reduced phage infectivity by ∼50%, stability experiments of the resulting MPs demonstrated a tolerance over a wide pH range (pH 2–10) as well as to oxidative (10 mm H_2_O_2_) and reducing conditions (100 mm glutathione) [69, 70].

**FIGURE 5:**
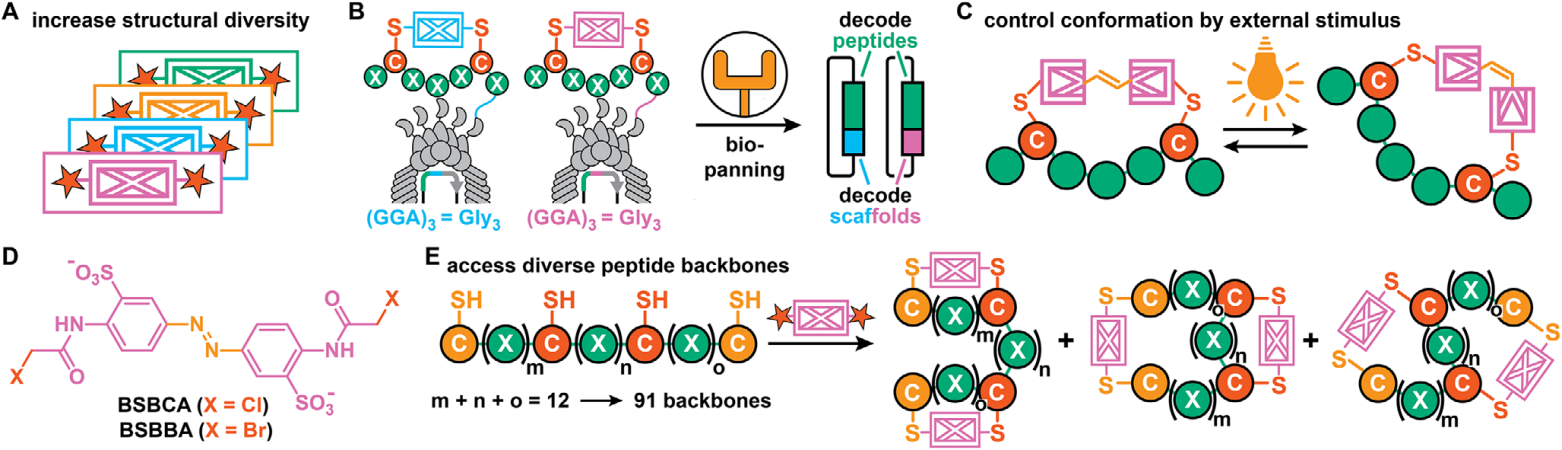
Increasing structural and conformational diversity of peptide libraries with cysteine crosslinkers. (A): Using a set of crosslinkers adds structural and functional diversity to hybrid MPs. (B): Genetic barcoding enables simultaneously selecting hybrid MPs library featuring different cyclization units. The identity of selected peptides and matching crosslinker can be identified by sequencing following biopanning. (C): Incorporation of photoswitchable cyclization units can exert control over the conformational state of hybrid MPs. (D): Structures of azobenzene‐base, photoswitchable crosslinkers employed in phage display. (E): The conformational diversity of bicyclic MPs can be increased by starting cyclization reactions with libraries featuring cysteine residues in varying positions. For example, crosslinking four cysteines with one cyclization unit, results in the formation of three distinct topologies.

Furthermore, the Pentelute group explored different arylation chemistries that can be used to crosslink cysteine residues under phage‐compatible conditions. Most notably, Spokoyny and coworkers demonstrated that hexafluorobenzene (**HFB,** Figure 4B) can undergo an S_N_Ar reaction with thiolates [71]. Further studies by Fadzen et al. showcased that the cyclization of synthetic peptides with **HFB** bestowed the resulting MPs with increased stability against proteases and the ability to traverse the blood‐brain‐barrier, thus highlighting the altered pharmacokinetic parameters of hybrid MPs [72]. However, perfluorinated arenes, such as **HFB**, suffer from slow reaction speeds and low solubility in water, which rendered them initially unsuitable for cyclizing linear precursors displayed on bacteriophages [73].

Recognizing their untapped potential, Kalhor‐Monfared and colleagues subsequently introduced a new generation of perfluoroarene cross‐linking agents, which featured a sulfone group to improve their solubility in aqueous buffers (**DFS**, Figure 4B) [74]. A study into the reactivity of these scaffolds confirmed that electron‐withdrawing substituents increased the reactivity of these compounds in water, inspiring further designs of crosslinkers by Zheng and coworkers (e.g. **DFB**, Figure 4B) [75]. The electron‐deficient nature of these scaffolds also stabilizes the resulting thioether linkages, making them less susceptible to oxidative degradation than those formed with **TBMB** [74, 75]. Moreover, the scaffolds employed by the Derda and Wu groups exhibited higher selectivity for cysteine residues, only showing modest cross‐reactivity with lysine side‐chains at pH 9. In contrast to analogous experiments with **TBMB**, mono‐substituted peptides could readily be obtained in absence of a second cysteine in peptide substrates. This improved selectivity likely also explains why phage infectivity is largely unaffected even in presence of high concentrations of perfluoroarene crosslinkers (1 mm **DFB** and 0.5 mm **DFS**). Wong and coworkers further showcased that the pharmacokinetic parameters of lead compounds identified from phage display campaigns with perfluoroarene crosslinkers can be finetuned [76]. Specifically, while albumin‐binding hybrid MPs containing **DFS** were prone to degradation in presence of glutathione, replacing the nonpeptidic moiety with a less reactive pentafluorophenyl sulfide improved in vivo circulation half‐time.

### 3.3 The Importance of Structural and Conformational Diversity

Phage‐displayed peptide libraries are inherently limited in size (∼10^9^ variants) due to bacterial transformation efficiencies. However, post‐translational cyclization with nonpeptidic linkers can significantly expand their structural and conformational diversity in three key ways (Figure 5): (1) scaffolds with distinct structures and functionalities can be employed in affinity selections; (2) reactive moieties within a given cyclization unit can provide control over MP conformations upon applying an external stimulus, such as UV‐light; and (3) varying the positions of multiple cysteine residues within a target peptide can give rise to (mono or) bicyclic peptides with distinct conformations. Over the past decade, a number of studies have demonstrated the power of such diversification and cyclization strategies to enrich selections of potent, hybrid MP‐binders.

In 2012, Chen and colleagues were the first to demonstrate that different cysteine cross‐linkers could impose distinct peptide‐backbone conformations on (bi)cyclic peptides (Figure 5A) [65]. Specifically, cyclizing one phage‐display library with different symmetrical crosslinkers gave rise to MPs with distinct sequences and binding affinities to a model target. While using a diverse set of cyclization units to increase structural diversity in the selections of hybrid MPs is attractive, this approach is also complicated by the fact that the identity of the scaffold is not genetically encoded. As a consequence, selections with different cyclization units have to be performed separately to enable the downstream structure deconvolution of hybrid MP binders.

A creative solution to this problem was introduced by Tjhung et al. in the form of *silent barcoding* [77]. In this strategy, the identity of a given cyclization unit is encoded by distinct codon usage in the invariable GSSG‐linker region that is used for fusing the peptide library to the pIII protein (Figure 5B). Thus, following cyclization reactions of individual phage libraries featuring these silent barcodes, all populations can be pooled and selections performed against a target of interest in parallel. Unfortunately, this strategy does not allow for multiple consecutive rounds of biopanning, as a mixture of phages with different silent barcodes is recovered, preventing attribution of enrichment to a specific scaffold. Critically though, deep sequencing of phages recovered after one round of biopanning has been shown to reveal consensus motifs of MP binders, thus (potentially) alleviating the need to perform additional rounds of biopanning [78].

Consequently, the use of silent barcodes has the potential to rapidly assess a diverse set of nonpeptidic cyclization units in the selection of hybrid MPs. In this regard, it is notable that the number of cyclization units has increased significantly over the past decade, including dimethylmaleimide (see Figure 4C for a small selection) [79, 80, 81]. Among these, an oxetane‐containing linker (**BBMO**) introduced by Martínez‐Sáez and coworkers in 2017 is potentially of particular interest, as **BBMO** has been shown to induce a sharper turn than previously explored scaffolds [82]. Consequently, a silent barcoding strategy employing structurally and functionally diverse scaffolds, such as **BBMO**, could identify hybrid MPs with a significantly‐broadened structural and conformational flexibility.

A more direct means to control conformations of selected hybrid MPs are photoswitchable cyclization units, which undergo significant structural changes upon irradiation with (UV) light (Figure 5C). Proof‐of‐concept for the selection of hybrid MPs, whose affinity can be controlled by light was provided by the Derda and Heinis groups in 2014, who used the azo‐benzene‐containing cyclization units **BSBCA** or **BSBBA** in their studies (Figure 5D) [83, 84]. Indeed, selections employing these photoswitchable crosslinkers elicited MP‐binders, whose affinity for a model target (streptavidin) was dependent on the E/Z isomerization state of the azobenzene unit. Specifically, the two studies led by Jafari and Bellotto identified photoswitchable MPs with 5‐10‐fold higher affinities in their active isomerization state, albeit the selected peptides did not display high binding affinities (*K*_D_ in the µm range). The need for symmetrical crosslinkers has thus far limited efforts toward this concept to these two examples. Likely, more versatile cyclization strategies (*vide infra*), which can incorporate asymmetric photoswitches that undergo larger structural change upon excitation with visible light will be required to assess the full impact of photoswitchable, hybrid MPs as chemical probes or application in photopharmacology [85].

A third way to increase the structural and conformational diversity of phage libraries is to vary the positions of cysteine residues, thereby creating mono‐ or bicyclic peptides with distinct patterns and conformations after cyclization (Figure 5E). This approach mirrors the creation of disulfide‐rich peptides, which form rigid and stable cores, conferring high proteolytic stability and binding efficiency. While these strategies do not incorporate nonpeptidic moieties for cyclization, the Wu group's design and selection of disulfide‐directed multicyclic peptides underscore the importance of conformational diversity in macrocyclic peptide selection [86]. The incorporation of nonpeptidic scaffolds in such efforts was first showcased by Kale and coworkers, who created two libraries of the formats XCX_3_ CX_3_ CX and XCX_4_ CX_4_ CX, which feature randomized amino acids (X) between three fixed cysteine residues [87]. Critically, the NNK codons employed for randomization ensured that ∼20%–25% of all library members featured a fourth cysteine residue at a variable position. As such, when incubated with a symmetrical bis‐electrophile, sequences featuring four thiol side‐chains could form three different bicyclic peptides, in which either cysteines 1–2 & 3–4, 1–3 & 2–4, or 1–4 & 2–3 are crosslinked (Figure 5E). Notably, cyclizing these libraries independently with six nonpeptidic scaffolds and performing biopanning cycles against three different targets, selected predominantly MPs with four cysteines present. Independent synthesis of all three double‐bridged peptides demonstrated that only one of the possible isomers displayed high affinities (low nM concentrations) against their target.

To not rely on randomization for the introduction of a fourth cysteine residue, Carle et al. subsequently constructed a total of 91 phage libraries, which encoded for all possible combinations of placing four reactive cysteines in an 18‐amino acid peptide (Figure 5E) [88]. Cyclization with one bis‐electrophile scaffold (or by disulfide formation), yielded an impressive structural diversity of 273 different bicyclic constructs—as four cysteines can be connected in three different ways. Intriguingly, model selections against human coagulation factor Xia revealed that MPs that displayed high affinities were preferentially elicited from only 3–4 out of the 91 libraries. This skewed distribution underscores how crucial structural and conformational diversity is for successful selections: only a small subset of backbone configurations yielded high‐affinity binders, suggesting that limited diversity in peptide topology may preclude the discovery of optimal ligands — especially for challenging therapeutic targets.

### 3.4 Diversifying Peptide Libraries Post‐Cyclization

Beyond their inherent chemical diversity and the conformational control they can exert, nonpeptidic cyclization units offer additional opportunities to chemically upgrade hybrid MPs. Specifically, bis‐electrophiles can be equipped with orthogonal reactive handles, enabling the *post‐cyclization* diversification of phage‐displayed libraries (Figure 6A) [89]. This approach facilitates the installation of pharmacophores, biophysical probes, or reactive warheads that would otherwise interfere with macrocyclization or phage viability. Suitable handles include aldehydes and ketones, whose reactivity is largely orthogonal to the nucleophilic substitution and conjugate addition chemistries used for cysteine crosslinking. Additionally, condensation reactions involving carbonyl groups are generally phage‐compatible, as evidenced by growing efforts to site‐selectively install and modify aldehydes and ketones on intact phage particles [90].

**FIGURE 6:**
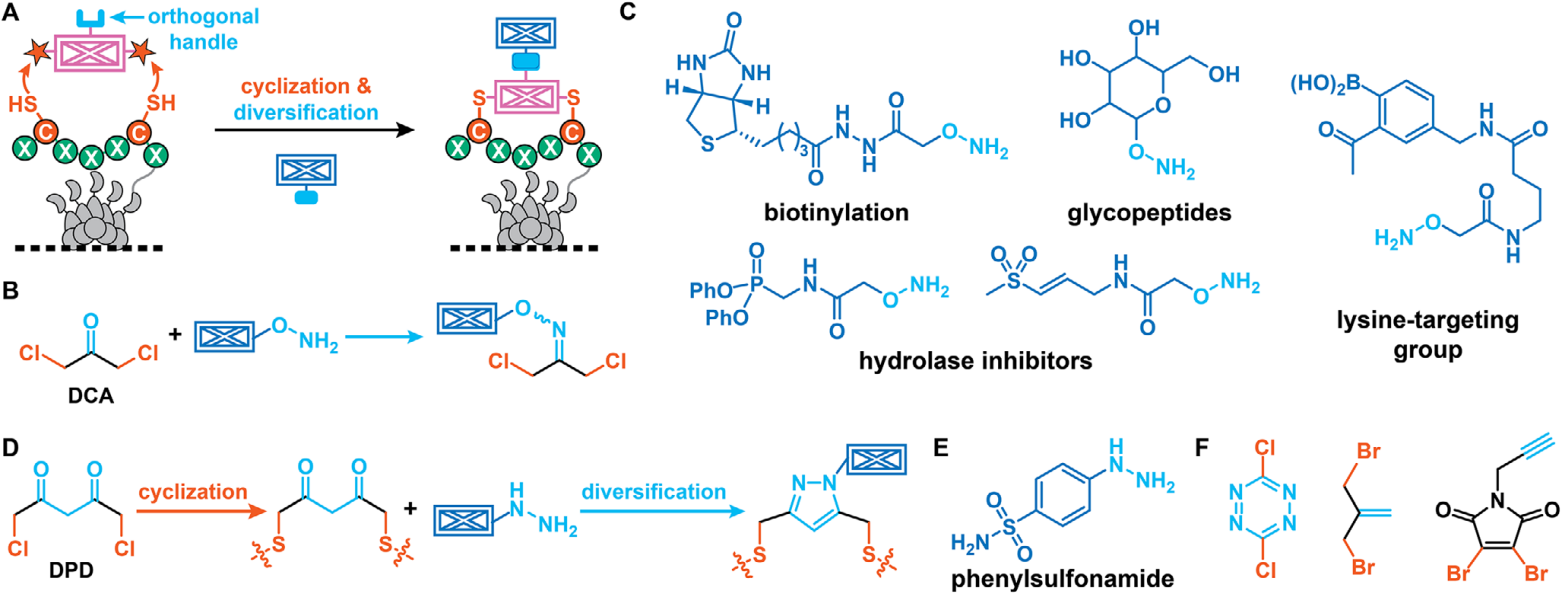
Pre‐ or post‐cyclization modification of symmetrical crosslinkers can install additional functionality into hybrid MPs. (A): Schematic representation of diversifying cyclization units featuring an orthogonal handle. (B): Condensation of DCA with oximes can expand the structural diversity of cysteine crosslinkers. (C): Structures of oximes used for the diversification of DCA. (D): A 1,3‐diketone handle can undergo a Knorr pyrazole synthesis post cyclization of peptides on bacteriophages. (E): Structure of the phenylsulfonamide used for post‐cyclization diversification. (F): Structures of symmetrical cysteine crosslinkers featuring orthogonal handles that have thus far remained unexplored in the selection of hybrid MPs.

Aldehydes, for example, are readily introduced on bacteriophages featuring an N‐terminal serine residue at pIII via the selective oxidation of 1,2‐aminoalcohols by periodate [91, 92]. Similarly, the Francis group could employ transamination reactions with pyridoxal 5’‐phosphate to install a ketone at the N‐terminus of the pVIII protein [93, 94]. Both aldehydes and ketones can undergo further condensation reactions with appropriate nucleophiles [91, 95], and have been employed to provide proof‐of‐principle for the derivatization of hybrid MPs post‐cyclization.

Toward this end, ketone‐functionalized, symmetrical crosslinkers are employed, with dichloroacetone (**DCA**, Figure 6B) providing a topical example. Inspired by pioneering studies from Assem and Derda [96], **DCA** was employed by the Derda group to cyclize cysteine‐flanked peptides on whole phages, introducing a central ketone suitable for oxime ligation [97]. Subsequent derivatization with aminooxy‐biotin demonstrated the feasibility of this approach, although reaction conditions (100 mM aniline acetate, pH 4.7) led to a >99.999% loss of phage infectivity, a critical limitation preventing downstream amplification.

To circumvent this issue, Ng and Derda pivoted to *pre‐functionalizing* **DCA** with chemically diverse oximes prior to peptide cyclization on whole bacteriophages. Specifically, they introduced a series of carbohydrates to create diverse glycopeptide libraries, although selections were not further pursued [97]. Since then, the Bogyo and Gao groups have employed **DCA**‐based scaffolds with reactive warheads as a modular platform to generate covalent inhibitors for enzymatic targets (Figure 6C). For this, Chen and colleagues installed a cysteine‐reactive vinyl sulfone and a serine‐reactive diphenylphosphonate to select MPs that irreversibly inhibit Tobacco etch virus protease and fluorophosphate‐binding hydrolase F, respectively, with nanomolar affinity [98]. Meanwhile, Zheng et al. employed **DCA** bearing 2‐acetylphenylboronic acid to reversibly bind lysine residues in sortase A from Staphylococcus aureus and the SARS‐CoV2 spike protein through iminoboronate formation (Figure 6C) [99].

Beyond **DCA**, other symmetrical crosslinkers have since enabled the diversification of MP libraries post‐cyclization. For example, employing the symmetrical 1,5‐dichloropentane‐2,4‐dione (**DPD**) as cyclization reagent, yielded 1,3‐diketone‐bearing MPs, which could be further condensed with diverse pharmacophores featuring a reactive hydrazine handle (Figure 6D) [100]. Importantly, EDTA rendered this Knorr pyrazole synthesis phage‐compatible, likely by chelating trace metals that might otherwise induce degradation or side reactions. Using this strategy, Ekanayake et al. upgraded hybrid MPs with zinc‐chelating phenylsulfonamides and selected nanomomlar binders against bovine carbonic anhydrase (Figure 6E).

Combined these examples demonstrate that the diversification of cyclization units in hybrid MPs represents a powerful means to identify high‐affinity (covalent) inhibitors. In the future, we anticipate that the *post‐cyclization* diversification strategy will benefit from a broader set of diversifiable scaffolds, such as the recently employed 3,5‐bis(bromomethyl)benzaldehyde (**KYL**, *vide infra*) [101]. In particular, we suggest that three additional symmetrical scaffolds, such as an alkyne‐bearing dibromo‐maleimide, dichlorotetrazine or a dibrominated isobutene derivate, merit evaluation in phage display (Figure 6E) [79, 102, 103]. While each of these scaffolds has been used in peptide stapling and are (reversibly) diversifiable, none of them has yet been explored in the context of phage display selections. We anticipate that their incorporation into phage‐compatible workflows will significantly expand the chemical versatility of hybrid MP libraries and facilitate the discovery of increasingly complex, functional peptide‐based binders

## 4 Phage‐Compatible Cyclization Strategies to Install Asymmetric Scaffolds

### 4.1 Considerations for the Programmed Modification of Two Distinct Residues

The structures of many potent natural and man‐made hybrid MPs often feature asymmetric, nonpeptidic moieties (Figure 1C–F), which are crucial for their biological activity. In contrast, the phage‐compatible cyclization strategies discussed in the previous section rely on crosslinkers with two‐ or three‐fold symmetry. This constraint arises as targeting two or more cysteine residues with asymmetric linkers typically results in regioisomeric mixtures of macrocycles. While selections using such mixtures are feasible, each isomer of a selected hit must be independently synthesized and evaluated, complicating downstream validation.

To avoid these complications and generate well‐defined hybrid MPs, cyclization with asymmetric scaffolds requires the programmed and selective modification of two distinct functional groups within the displayed linear peptide (Figure 7A). However, given the stringent requirements for both reactivity and selectivity (*cf*. Section 3.1), only a few such strategies have been reported over the past five years. Notably, all involve a combination of cysteine alkylation with a chemical or enzymatic reaction targeting the N‐terminus. As was the case for the application of cysteine crosslinkers, these strategies have built on pioneering efforts to selectively modify protein N‐termini under mild, phage‐compatible conditions [104, 105, 106, 107].

**FIGURE 7:**
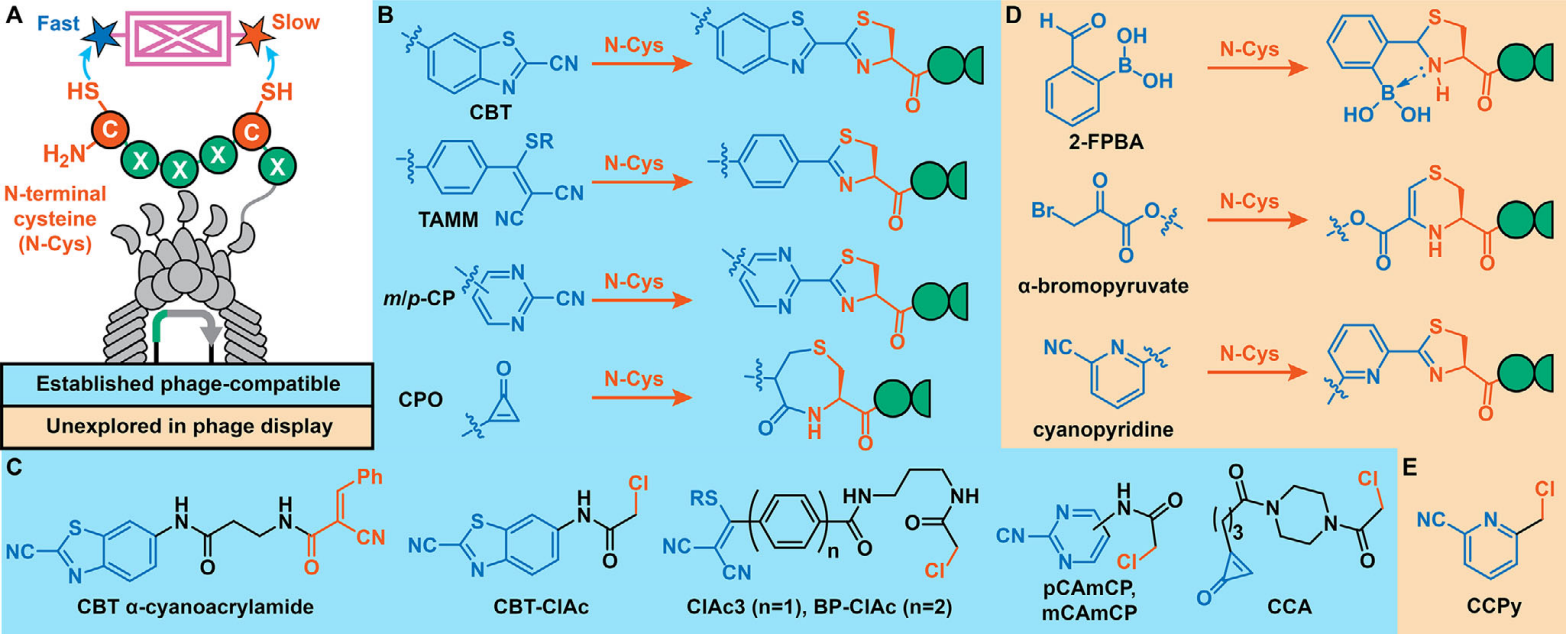
Phage‐compatible macrocyclization strategies targeting N‐terminal and internal cysteines. (A): Schematic representation of incorporating asymmetric, nonpeptidic scaffolds by taking advantage of the differential reactivities and kinetics of N‐terminal and internal cysteine residues. (B): Reactive groups that undergo selective condensation with the 1,2‐aminothiol moiety present in N‐terminal cysteines. C: Structures of asymmetric cyclization units used in phage‐display selections of hybrid MPs. D: Reactions of functional groups that selectively target N‐terminal cysteines, but have not been employed in phage‐display campaigns. E: Structure of an asymmetric cyclization reagent that remains unexplored in the selection of hybrid MPs.

### 4.2 Selectively Targeting N‐Terminal and Internal Cysteines

While traditional alkylation reactions are unable to differentiate between two cysteines, the presence of a 1,2‐aminothiol group at N‐terminal cysteine residues shows a distinct reactivity profile (Figure 7A) [108]. For example, the chemo‐selectivity observed in reaction with thioesters, aromatic nitriles, or aldehydes all attest on the unique reactivity of N‐terminal cysteine residues when compared to internal thiol side‐chains [109]. With available modification strategies that selectively target 1,2‐aminothiols steadily increasing, they have recently been applied for the selection of hybrid MPs featuring asymmetric scaffolds.

For example, Ren and coworkers originally developed a bio‐compatible nitrile‐aminothiol (NAT) “click” reaction between N‐terminal cysteines and 2‐cyanobenzothiazole (**CBT**, Figure 7B) derivatives for the labeling of proteins in vivo [110]. Following the application of this chemistry to peptide cyclization reactions in solution [111], both the Gao and Liu groups demonstrated its applicability for the selection of hybrid MPs featuring asymmetric cyclization units by bacteriophage display [112, 113]. While both research groups employed the **CBT** moiety to selectively modify an N‐terminal cysteine, they chose distinct second, reactive handles to ensure selective cyclization with their bi‐functional scaffolds.

To ensure macrocycles are only stable following the irreversible NAT reaction, Zheng, Haeffner, and Gao selected an α‐cyanoacrylamide group [114], which exhibits fast but reversible thiol conjugation kinetics (Figure 7C). Despite this inherent reversibility, the formed MPs proved sable in aqueous buffer upon addition of cysteine (250 µm) and in presence of 1 mM glutathione (GSH : GSSG, 9 : 1) [113]. However, partially trapping of free cysteines was observed by addition of iodoacetamide (500 µm over 12 h), albeit at a rate ∼1000‐fold slower than the one observed in the reaction of α‐cyanoacrylamide with linear peptides. Notably, this programmed cyclization proved compatible with phage display and enabled the selection of hybrid MPs with low nano‐ and micro‐molar affinity for Keap1 and sortase A, respectively [113].

In addition to the CBT group, Hampton and coworkers equipped their bi‐functional scaffold with a chloroacetamide moiety, which irreversibly alkylates thiols ∼450‐fold slower than the corresponding NAT reaction (Figure 7C) [112]. Consequently, treating peptides with this bifunctional scaffold yields MPs following the rapid condensation between **CBT** and the N‐terminal cysteine and the proximity‐driven alkylation of an internal thiol by weakly‐electrophilic chloroacetamide. The bifunctional, asymmetric scaffold, **CBT‐ClAc**, did not negatively impact phage infectivity at 100 µm, but was able to modify 60% of phages as determined by a pull‐down experiment. Applying this cyclization strategy in phage display selections against epitopes of the SARS‐CoV‐2 spike protein elicited hybrid MP binders with single digit micromolar affinity.

Since these initial reports, taking advantage of differential kinetics between thiol modification reactions has proven a fruitful strategy for the programmed modification of N‐terminal and internal cysteine residues. For example, the Wu group first described 2‐((alkylthio)(aryl)methylene)malono‐nitrile (**TAMM**) as a fast and selective reagent for N‐terminal cysteines (Figure 7B) [115]. In this transformation, **TAMM** undergoes a multi‐step cascade reaction that is triggered by vinyl‐sulfide exchange and yields stable 2‐aryl‐4,5‐dihydrothiazoles. To enable the cyclization of linear peptides displayed on bacteriophages, Zheng and colleagues constructed an asymmetric scaffold (**ClAc3**), featuring both the **TAMM** moiety and the less reactive, chloroacetyl group (Figure 7C) [115]. Showing excellent phage‐compatibility—no discernable effect on infectivity with up to 300 µm was observed—the bifunctional **ClAc3** was successfully employed in selection of hybrid MP binders with sub‐micromolar affinity for Bcl‐2, Keap1, and Mdm2. Notably, this strategy was also recently applied by Liu et al. to elicit hybrid MPs that feature a rigid biphenyl‐moiety (**BP‐ClAc**, Figure 7C) [116]. Following the selective cyclization on phages, the group identified hybrid MPs that bind the potential cancer‐target BCL‐X_L_ with low, nanomolar affinities.

To address potential concerns with respect to synthetic accessibility, side‐reactivity, and/or the introduction of a large *chemical scar* in the formed hybrid MPs, two recent reports have paired chloroacetamide moieties with smaller reactive groups that selectively target N‐terminal cysteines. For example, Hampton and coworkers made use of electron‐deficient 2‐cyanopyrimidines to provide stable thiazolidine adducts (Figure 7B) [117]. From five derivatives, 2‐chloro‐N‐(2‐cyanopyrimidin‐5‐yl)acetamide (**pCAmCP**) and 2‐chloro‐N‐(2‐cyanopyrimidin‐4‐yl)acetamide (**mCAmCP**) emerged as efficient nonpeptidic scaffolds to cyclize phage‐displayed peptide libraries containing an N‐terminal and internal cysteine (Figure 7C). Notably, the strategy enabled the selection of hybrid MPs with nanomolar affinities against the ZNRF3 extracellular domain.

Additionally, the Bernardes group recently introduced cyclopropenone (**CPO**) reagents as an alternative to thiazolidine‐forming reagents, such as **TAMM, CBT,** or **m/p‐CAmCP**. Under mild and phage‐compatible conditions, **CPO** proved to react fast (k  =  67 m– ^1^ s–^1^) and selective with 1,2‐aminothiol group to yield stable 1,4‐thiazepan‐5‐one linkages (Figure 7B) [118]. Appending a chloroacetamide to **CPO** yielded the bifunctional scaffold, **CCA**, which enabled the efficient and selective cyclization of displayed peptides without impacting phage infectivity (Figure 7C) [119]. Critically, **CCA** could be employed in phage display selections, resulting in the identification of streptavidin‐ and integrin αvβ3‐binding hybrid MPs.

As the studies in this section attest, targeting N‐terminal cysteine residues to employ asymmetric, nonpeptidic scaffolds for the selection of hybrid MPs has already proven a versatile strategy. Nevertheless, future efforts will likely build on these initial successes by focusing on (1) thus far unexplored 1,2‐aminothiol modifying chemistries and (2) minimizing the *chemical scar* introduced by the employed cyclization reagents. For the former, the condensation of aldehydes with N‐terminal cysteines to form stable thiazolidines is an unexplored yet potentially a powerful application for the selection of hybrid MPs [120, 121]. Efforts have thus far fallen short, as these reactions typically suffer from slow kinetics and the need for acidic pH to proceed. Notably though, the Gao and Gois groups independently demonstrated that imine formation—the rate‐determining step in the thiazolidine formation—is readily accelerated by introducing an *o*‐boronic acid moiety (rate constant ∼10^3^ M^−1^ s^−1^) [122, 123]. Interestingly, MPs featuring such a 2‐formyl phenylboronic acid (**2‐FPBA**) structure exhibited improved chemical stability due to the presence of a B─N dative bond (Figure 7D), making bifunctional scaffolds based on **2‐FPBA** an attractive means for the future selection of hybrid MPs.

Another important factor for future engineers of hybrid MPs is to minimize the *chemical scar* that is introduced by the employed asymmetric scaffold. For example, while CBT groups react fast and selectively with N‐terminal cysteines, the *d*‐luciferin‐moiety produced could potentially compromise the pharmacological properties of selected peptides. Another advantage of small and stable reactive handles is improved synthetic accessibility, as it is highly desirable to construct a diverse panel of nonpeptidic cyclization units (cf. Section 3.3). In this context, two recent reports from the Nitsche group are worth highlighting, as they could prove impactful for future phage display campaigns. For once, Morewood and Nitsche reported that α‐bromopyruvate moieties selectively condense with 1,2‐aminothiols to form lanthionine‐ketenamine esters (Figure 7D) [124]. Notably, these moieties have been found in bioactive, naturally‐occurring lanthipeptides, which is indicative of the biological relevance of this motif [125]. Additionally, Ullrich and colleagues recently introduced 2‐chloro‐6‐cyanopyridine (**CCPy**, Figure 7E) as a simple bifunctional scaffold that can site‐selectively crosslink N‐terminal and internal cysteine residues in proteins [126]. Akin to **CBT,** cyanopyrimidines, and **TAMM** reagents, the cyanopyridine‐moiety undergoes condensation with the 1,2‐aminthiol to form a thiazoline moiety (Figure 7D). Being a small and synthetically‐accessible motif, it is conceivable that a library of asymmetric scaffolds featuring cyanopyridines and weakly reactive electrophiles (e.g. chloroacetamides) could soon find wide application in the phage display of hybrid MPs.

### 4.3 Exploiting the Unique Reactivity of N‐Terminal Amines

Selective modification of N‐terminal cysteines is enabled by the distinctive chemistry of N‐terminal α‐amines, which can cooperatively react with side‐chain functional groups on the first residue (e.g. in 1,2‐aminothiols). More broadly, N‐terminal α‐amines are particularly attractive functional groups for peptide and protein modification. Their proximity to the backbone carbonyl lowers their basicity (p*K*_a_ ≈ 7–9) relative to the ε‐amines of lysine side chains (p*K*_a_ ≈ 10.5, Figure 8A), enhancing their nucleophilicity under near‐neutral pH [104, 105]. This property is evident in several contexts: (1) classical amine‐targeting reagents such as *N*‐hydroxysuccinimide (NHS) esters and aldehydes preferentially react with N‐terminal amines at mildly acidic pH; (2) in absence of an appropriate number of thiols, symmetrical cross‐linkers, such as **BBMB** or **TBMB** (cf. Section 3.2) can undergo (bi)cyclization via alkylation of the α‐amine [61]; and (3) in cyclization approaches that target ε‐amines of lysine residues, the N‐terminus is typically acetylated during SPPS to avoid competing / dominant cyclization via the reactive α‐amine.

**FIGURE 8:**
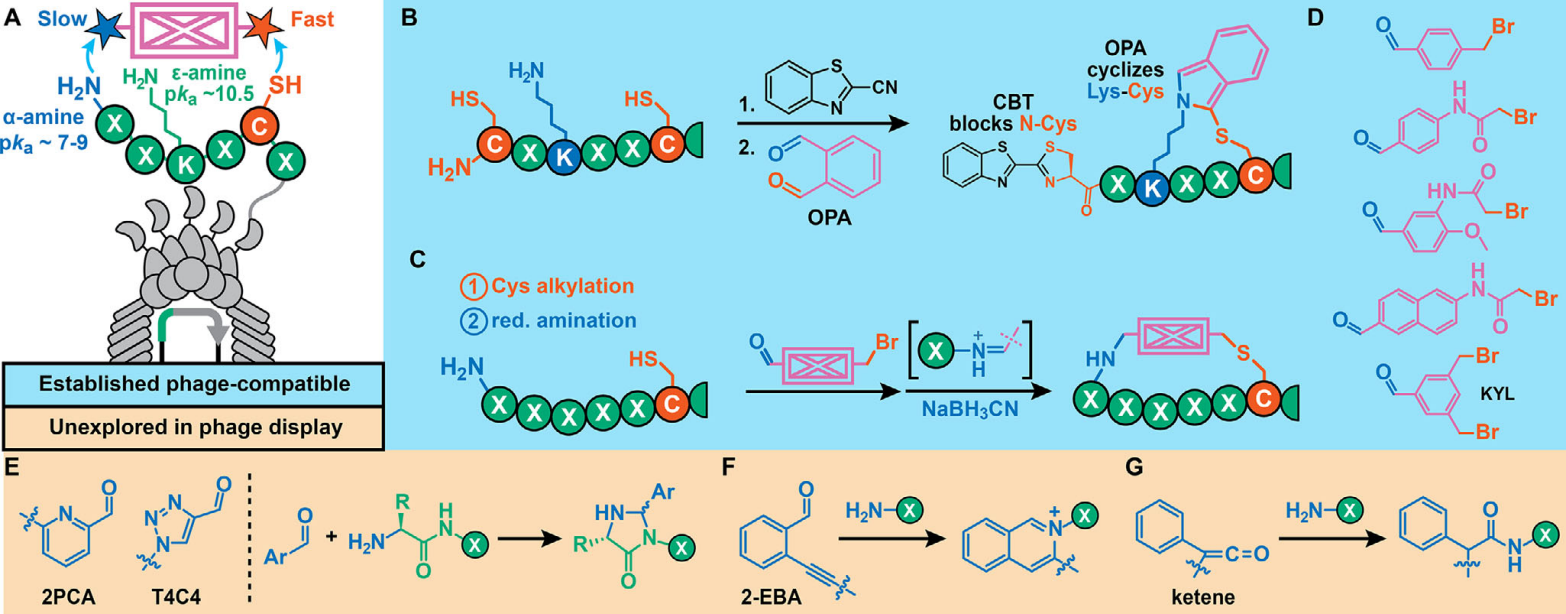
Phage‐compatible macrocyclization strategies using asymmetric scaffolds to target N‐terminal amines and internal cysteines. (A): Schematic representation of cyclization strategies exploiting the enhanced nucleophilicity of N‐terminal amines relative to lysine residues. (B): Blocking an N‐terminal cysteine with CBT, enables the selective cyclization of lysine amines and an internal cysteine with OPA. (C): Bifunctional scaffolds, featuring both an aldehyde and an alkylating group, facilitate a two‐step, one‐pot cyclization of phage‐displayed peptide libraries. (D): Structures of asymmetric, nonpeptidic scaffolds utilized in the phage display campaigns for the selection of hybrid MPs. (E,F): Functional groups that selectively target N‐terminal amines, but have not yet been employed in phage‐display campaigns.

For example, blocking the N‐terminus enables efficient macrocyclization via condensation of o‐phthalaldehyde (**OPA**) with cysteine and lysine residues, yielding fluorescent isoindole‐containing MPs [127, 128]. Using various **OPA** analogs, the Li and Perrin groups created mono‐ and bicyclic peptides, including a derivative of the toxic natural product α‐amanitin. Unfortunately, the lack of selectivity for lysine ε‐amines over α‐amines has hindered the direct application of **OPA** to phage display. To address this challenge, Xiang and coworkers developed a clever blocking strategy, selectively modifying the N‐terminal cysteine with **CBT** prior to **OPA**‐mediated cyclization (Figure 8B) [129]. This strategy enabled peptide libraries on phage, bearing both cysteine and lysine residues, to be cyclized with **OPA**, leading to the identification of hybrid MPs with micromolar affinity for therapeutically relevant targets (PTP1B, NEK7, and hKeap1). While this approach is likely compatible with **OPA** analogs [127, 128] and related reagents such as thiophene‐2,3‐dialdehyde [130], the use of asymmetric cross‐linkers will result in regioisomeric mixtures requiring post‐selection deconvolution.

Instead of blocking the N‐terminus, Oppewal and colleagues directly leveraged the increased nucleophilicity of the α‐amine to enable selective incorporation of asymmetric scaffolds [131]. Toward this end, they employed bifunctional cyclization units that combine a cysteine‐targeting electrophile with an aromatic aldehyde (Figure 8C,D). Following the initial cysteine‐alkylation step, macrocyclization was enabled via the proximity‐driven reductive amination in the presence of NaBH_3_CN (Figure 8C). Performing this two‐step procedure at mildly acidic pH ensured selectivity over lysine ε‐amines. Excitingly, this strategy also proved fully phage‐compatible and yielded high‐affinity (low nanomolar) binders for streptavidin.

Further highlighting the utility of proximity‐driven amine chemistry, Yan and colleagues employed the trifunctional scaffold 3,5‐bis(bromomethyl)‐benzaldehyde (**KYL**) for the *late‐stage reshaping* of pre‐selected phage‐displayed libraries (Figure 8D) [101]. Starting with a phage‐displayed library cyclized via disulfide bonds, they performed three rounds of biopanning against NS3aH1 protease. The resulting enriched peptide library was subsequently subjected to an additional selection step, in which peptides were cyclized with **KYL** by reductive amination. Notably, this late‐stage reshaping strategy, identified peptide sequences that maintained their nanomolar affinity for the target, while replacing a labile disulfide with stable thioether/secondary amine linkages.

Looking ahead, we anticipate growing interest in N‐terminal α‐amine‐directed cyclization strategies, as more potentially phage‐compatible labelling reactions have recently been described. For example, 2‐pyridinecarboxaldehyde (**2PCA**, Figure 8E) can condense with the α‐amine and the adjacent amide nitrogen to form stable imidazolidinones at physiological pH (phosphate buffer, pH 7.4) [132]. Related chemistry using triazole‐4‐carbaldehyde (**T4CA**, Figure 8E) has also been demonstrated for peptide cyclization, although requiring mixed organic solvents (DMF/H_2_O) and DMAP as a catalyst [133]. A different mode of activation was reported by Deng and coworkers, who introduced 2‐ethynylbenzaldehyde (**2‐EBA**, Figure 8F), which reacts with N‐terminal amines to form isoquinolinium species via 6‐endo‐dig cyclization [134]. Lastly, Chan and colleagues introduced ketene reagents for selective α‐amine modification, resulting in amide linkages (Figure 8G) [135]. Together, these studies highlight the underexplored potential of proximity‐driven reactions at the N‐terminal α‐amine for accessing hybrid MPs with asymmetric scaffolds in phage display systems.

### 4.4 Harnessing Enzymes for Residue‐Specific Cyclization Strategies

Compared to synthetic reagents, enzymatic transformations often meet the stringent requirements for site‐specific modification of peptides and proteins. They operate under mild, aqueous conditions, display high selectivity, and exhibit substantial rate accelerations, features that are particularly attractive for modifying dilute phage‐displayed peptide libraries [136, 137]. However, many enzymes routinely used in bioconjugation are poorly suited for generating hybrid MPs in phage display selections. Limitations that restrict their applicability include (1) reliance on activated ester substrates, (2) strict peptide length constraints, or (3) the need for well‐defined amino acid recognition motifs.

Despite these challenges, recent studies have highlighted the promise of enzymatic macrocyclization in the context of phage display. For example, two strategies have repurposed cyclases from ribosomally synthesized and post‐translationally modified peptide (RiPP) pathways for the enzymatic production of lanthipeptides, a diverse class of bioactive MPs featuring (methyl)lanthionine rings (Figure 9A) [138]. These efforts were enabled by studies from the van der Donk group, who characterized a number of highly promiscuous lanthipeptide synthases (e.g. ProcM) that could efficiently modify diverse serine/threonine‐ and cysteine‐containing core peptides, provided a conserved leader sequence is retained [139, 140].

**FIGURE 9:**
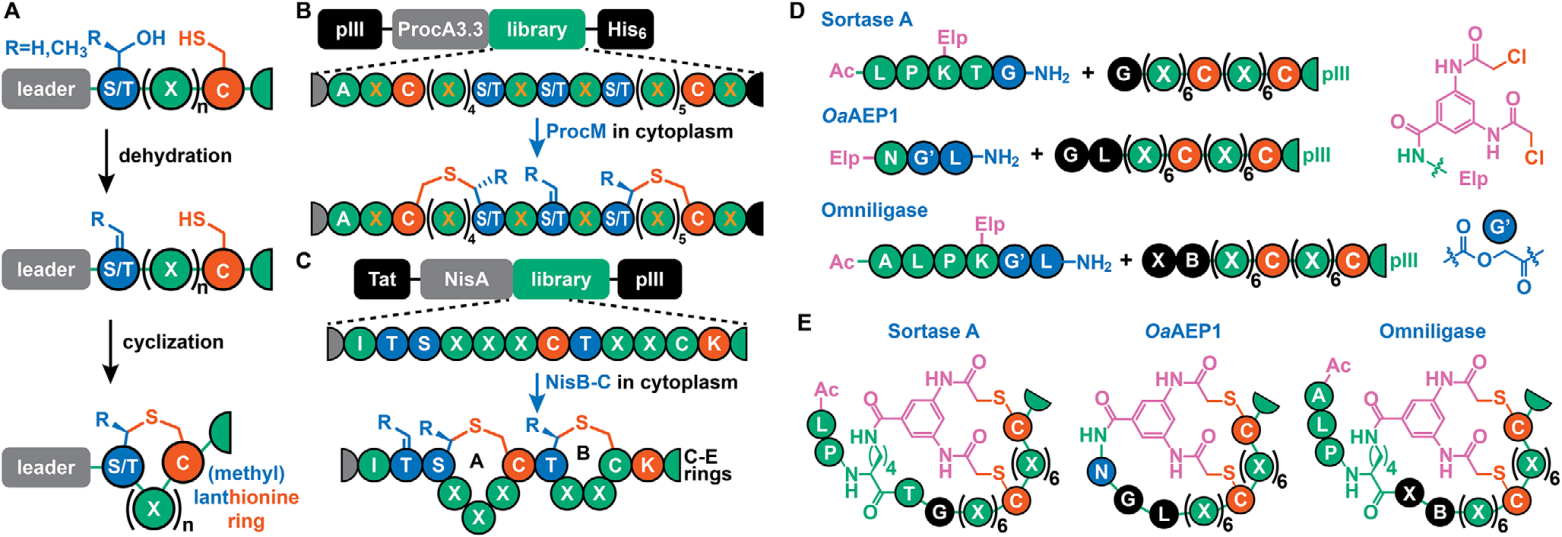
Phage‐compatible strategies that employ enzyme(‐mediated) cyclization for the selection of hybrid MPs. (A): Schematic overview of the biosynthesis of (methyl)lanthionine rings in lanthipeptides. (B): C‐terminal phage display of lanthipeptide libraries by producing ProcM in *E. coli* cytoplasm. (C): N‐terminal display of nisin libraries by taking advantage of the Tat export signal and the cytoplasmic production of NisB‐C. (D): Employing Sortase A, *Oa*AEP1, or omniligase for the ligation of Elp‐functionalized peptides to pIII‐displayed peptide libraries. (E): Hybrid MPs that result from cysteine alkylation following ligase‐mediated modification reactions.

Building on these findings, Urban and coworkers established a workflow for de novo selection of functional lanthipeptides by phage display [141]. A C‐terminal display strategy of pIII‐fused peptides proved critical, to increase the cytoplasmic residence time and allow for cyclization by co‐expressed ProcM and assembly into functional phages (Figure 9B). Using this approach, selections with naive lanthipeptide libraries yielded urokinase plasminogen activator (uPA)‐ and streptavidin‐binding peptides.

In parallel, Hetrick and colleagues also demonstrated the successful N‐terminal display of nisin derivatives, a potent antimicrobial lanthipeptide (Figure 9C) [142]. In addition to the co‐expression of Nisin‐cyclization enzymes, rerouting pIII to the tat export system proved crucial to extend the residence time of the linear precursors in the cytoplasm. This optimized system enabled the selection of lipid II‐binding variants from nisin libraries with randomized A‐ and B‐rings.

While both studies focused on in vivo peptide cyclization during phage assembly, RiPP biosynthesis enzymes could also lend themselves toward peptide cyclization and modification in vitro after phage isolation. A likely candidate for such developments is the lactazole RiPP machinery, which has recently been applied by the Suga group for the *de novo* discovery of thiopeptides by mRNA display [143, 144, 145].

Beyond RiPP pathways, several other classes of peptide‐modifying enzymes have been explored for in vitro macrocyclization [146]. For example, nonribosomal peptide cyclases (e.g. SurE), transpeptidases (e.g. sortase A), endopeptidases (e.g. butelase or OaAEP1), and engineered subtilisin variants (peptilligase or omniligase) have been used to cyclize peptide and proteins in vitro. Yet, these enzymes predominantly perform head‐to‐tail ligations, which are suboptimal for generating head‐to‐side or side‐to‐side macrocyclic topologies required for phage‐displayed peptides. To circumvent these limitations and enable the incorporation of nonpeptidic moieties into hybrid MPs, the Fang lab has recently pioneered chemoenzymatic strategies that combine enzymatic ligation with site‐specific chemical modifications.

Specifically, these reports employ ligases to attach peptides functionalized with a C2‐symmetric electrophile (**Elp**) to the N‐terminus of randomized pIII‐displayed peptide libraries (Figure 9D) [147, 148, 149]. Subsequent intramolecular substitution by two downstream cysteine residues yields stable bicyclic peptides featuring both thioether and amide linkages (Figure 9E). Depending on the ligase used, these methods depend on short recognition sequences, which remain as fixed *scars* in the bicyclic peptides. While these recognition sequences are invariable for Sortase A (Ac‐NH‐LPKTG) and *Oa*AEP1 (NH‐NGL) [147, 148], a recent report making use of omniligase alleviated some of these constraints (Figure 9D,E). Indeed, Wan and coworkers confirmed that the broad substrate promiscuity of omniligase enables the efficient cyclization of 130/400 dipeptide sequences following the ligation site [149]. Notably, the Fang lab demonstrated that either of these three strategies were compatible with phage display, enabling them to select multiple binders for TEAD4.

Although the need for recognition sequences remains a key limitation in the modification and cyclization of fully random peptide libraries, these examples highlight the potential of carefully‐designed chemoenzymatic workflows. With protein engineering efforts ideally suited to expand the substrate scope and alter recognition motifs of ligases and cyclases [150, 151], (chemo)enzymatic transformations are poised to play a central role in the future selection of hybrid MPs by phage display.

## 5 Off the Beaten Paths—Un(Der)Explored Strategies for the Selection of Hybrid MPs

While enzymatic and chemical strategies have already expanded the design space for phage‐displayed hybrid MPs, a vast number of chemistries remain un(der)explored. As shown above, classic protein labelling chemistries with selectivity for cysteine, lysine, and N‐terminal amines have dominated strategies to cyclize peptides on phages. However, the reliance on symmetrical cross‐linkers or highly specialized reactive moieties means that the majority of the used cyclization units are more limited in structural diversity than those found in natural products. Critically though, a wide arsenal of reported peptide cyclization chemistries remains unexplored for cyclizing peptides on phages. Given their mild reaction conditions, such strategies are likely phage‐compatible, and, consequently, could open up new avenues for selection of natural‐product‐like, hybrid MPs. The following sections provide a concise overview of approaches that we surmise could find application in biological selections in the (near) future.

### 5.1 Expanding the Residue‐Specific Toolkit for Phage‐Compatible Macrocyclization

Thiols and amines have long served as the primary handles for peptide modification and cyclization in phage display. However, recent advances in bioconjugation have expanded the chemical toolbox, enabling selective targeting of a broader range of amino acid side chains under mild, aqueous conditions [53, 152, 153]. Many of these strategies, originally developed for protein labeling or chemical proteomics, show promising compatibility with unprotected oligopeptides and could open up new avenues for phage‐compatible macrocyclization. This section highlights (1) emerging methods that modify less commonly targeted residues, (2) proximity‐driven chemistries that enable residue‐selective modifications in complex environments, and (3) the unique potential of N‐terminal proline residues as macrocyclization handles (Figure 10A).

**FIGURE 10:**
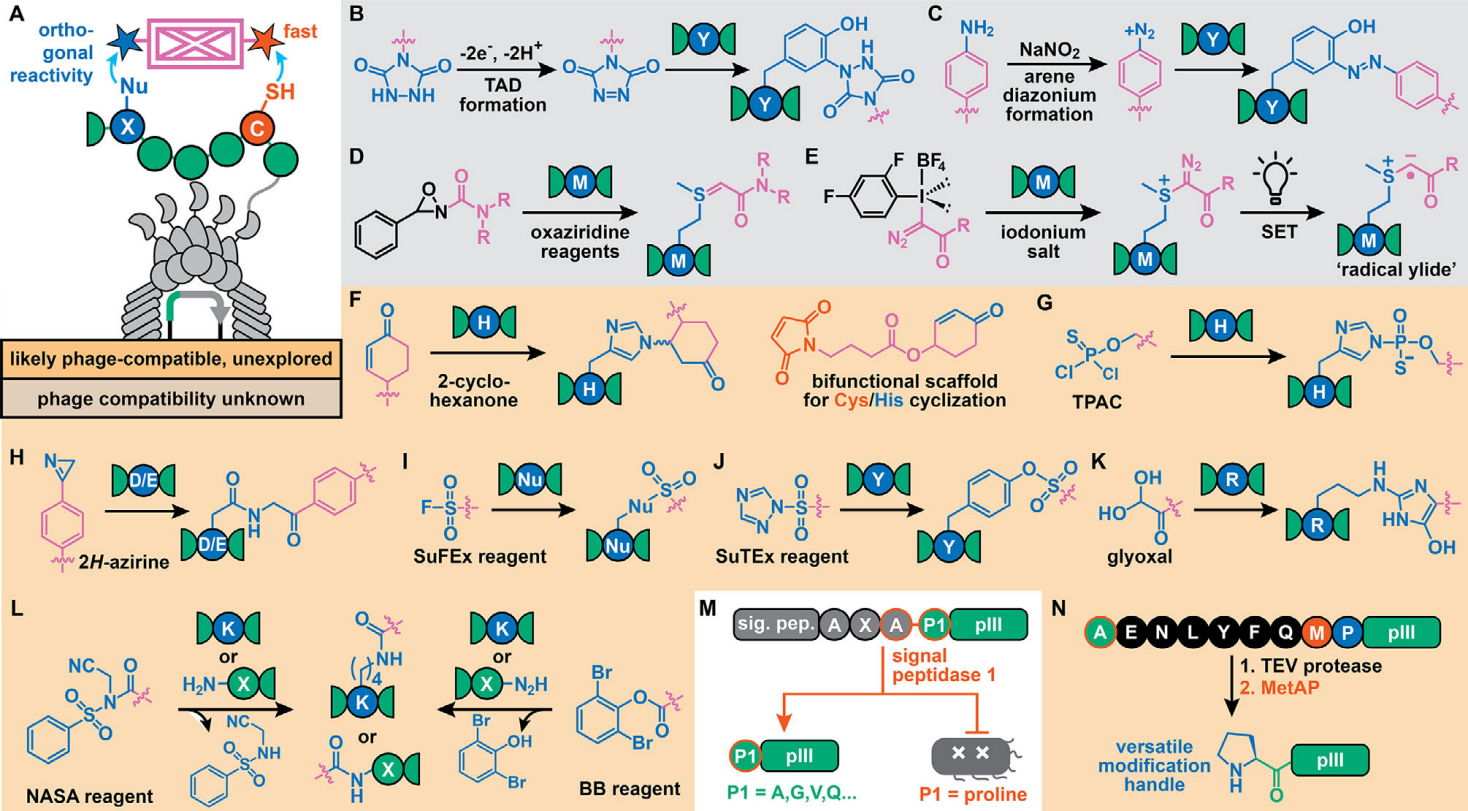
Residue‐specific modification reactions un(der)explored in phage display of hybrid MPs. (A): Schematic representation of theoretical on‐phage peptide cyclization using a combination of fast cysteine alkylation chemistry combined with orthogonal reactivity targeting side chains of other amino acids or the N‐terminus. (B,C): Structures of reagents which target tyrosine residues. (D,E): Structures of reagents which selectively modify methionine residues. (F,G): Structures of biocompatible reagents which selectively modify histidine residues. (H): Structures of biocompatible reagents which selectively modify carboxylic acid‐containing amino acid residues. (I): Biocompatible sulfur‐fluoride exchange chemistry targeting nucleophilic amino acid residues. (J): Biocompatible sulfur‐triazole exchange chemistry targeting tyrosine residues. (K): Glyoxal reagents for selective modification of arginine side chains in peptides and proteins. (L): Weak acylating reagents that undergo proximity‐driven reactions with N‐terminal or ε‐amines of lysine side chains. (M): In order to obtain phages displaying peptides featuring an N‐terminal proline, engineering of signal peptidase I (SP1) to process leader sequences featuring a proline in the +1 position can be done via growth selection of SP1 variants. (N): A two‐step strategy to generate an N‐terminal proline on phage using cleavage with TEV protease (1) followed by removal of the N‐terminal methionine using methionine aminopeptidase (2).

#### 5.1.1 Targeting Tyrosine Residues

The phenolic side chain of tyrosine offers a unique reactivity profile and is often buried within folded proteins. These characteristics make tyrosine an attractive candidate for site‐selective cyclization when introduced in randomized, phage‐displayed peptide libraries. For example, Ban and coworkers pioneered the use of N4‐substituted 1,2,4‐triazoline‐3,5‐dione (**TAD**) reagents, which undergo rapid, selective electrophilic aromatic substitution with tyrosine—but not tryptophan—in aqueous buffer (Figure 10B) [154, 155]. Building on these findings, Keyes and colleagues developed a synthetic strategy to access linear peptides containing masked **TAD** precursors and a single tyrosine residue [156]. Mild oxidation (e.g. using NaNO_2_) could activate the precursor and generate diverse MPs via reaction with the tyrosine phenol. While employing NaNO_2_ led to oxidations at cysteine, methionine, and tryptophan residues, generating the reactive **TAD** species electrochemically, might be used in phage display selections to prevent these side reactions [157].

Tyrosine‐selective arylation has further been achieved using arene diazonium salts (Figure 10C). These reagents have been used to modify interior tyrosine residues of the bacteriophage MS2 capsids, highlighting their potential application to whole‐bacteriophage systems [158]. Notably, only electron‐deficient diazonium reagents display fast tyrosine‐modification kinetics, providing a straightforward tool to tune their reactivity for cyclization reactions. Additionally, although diazonium salts can arylate cysteine thiols, their poor reactivity under physiological conditions reduces the risk of off‐target labelling [159]. As such, it is conceivable that both **TAD** and arene diazonium salts could be incorporated into bifunctional scaffolds featuring cysteine‐reactive electrophiles. Akin to strategies presented in Section 4, macrocyclization could be achieved by the programmed modification of a thiol and a nearby phenol side chain.

#### 5.1.2 Targeting Methionine Residues

Due to its susceptibility to oxidation, methionine (and thioethers in general) are typically excluded from therapeutic peptides. However, Lin and colleagues leveraged this sensitivity to develop an oxidative sulfur imidation reaction using oxaziridine‐based reagents (Figure 10D) [160]. Their Redox Activated Chemical Tagging (ReACT) strategy enabled selective methionine modification in peptides and proteins, with cysteine oxidation to cystine as the only observed cross‐reactivity.

Despite the abundance of multiple nucleophilic side chains in proteins [161], Taylor and colleagues also reported the electrophilic modification of methionine residues [162], By fine‐tuning the reactivity of a iodonium salt to target the polarizable thioether moiety, they could convert thioethers into cationic sulfonium species (Figure 10E). The presence of an electron‐deficient arene and a tetrafluoroborate as counterion proved crucial to extend the half‐life of the reagent in water and give rise to good conversion (>95%) in presence of 20 mm thiourea.

Unlike oxaziridine reagents, which oxidize cysteines to cystines, iodonium salts are prone to alkylating free thiols. Nevertheless, when incorporated into bifunctional scaffolds featuring cysteine‐alkylating groups, both reagent classes are potentially compatible with hybrid MP formation on bacteriophages. Additionally, the diazo sulfonium conjugates formed by iodonium salts can undergo further functionalization via single‐electron transfer photocatalysis (Figure 10E), providing a potential route for post‐cyclization modifications of hybrid MPs.

#### 5.1.3 Targeting Histidine, Glutamate, and Aspartate Residues

While less commonly exploited, we would like to highlight a selection of recently reported reagents that target alternative nucleophilic residues. For instance, Joshi and Rai introduced 2‐cyclohexenone as a synthetically accessible and readily modifiable electrophile with modest selectivity for histidine (Figure 10F) [163], Its potential for programmed protein/peptide cyclization was further demonstrated by Wu and coworkers, who employed a maleimide‐cyclohexenone scaffold as a proximity‐driven cysteine–histidine crosslinker (Figure 10F) [164]. Additionally, Jia and colleagues developed an alternative approach for histidine modification, which was inspired by naturally occurring histidine phosphorylation. Specifically, they introduced thiophosphoroalkyne dichloridate (**TPAC**) as a phosphorus‐based electrophile capable of selective histidine conjugation (Figure 10G) [165].

Lastly, carboxylates, found in glutamate, aspartate, and at the peptide C‐terminus, also represent potentially underutilized targets for macrocyclization. From the available strategies, 2*H*‐azirine‐based reagents have shown promise for selective modification of carboxylates under mild conditions, forming stable amide bonds in the resulting products (Figure 10H) [166]. These characteristics make bifunctional cyclization units featuring 2*H*‐azirines an attractive choice for exploring the cyclization of C‐terminal peptide‐pIII fusions.

#### 5.1.4 Proximity‐Driven Reactions

Over the past few decades, chemical proteomics has mapped and interrogated a wide range of protein–small molecule interactions in complex cellular environments [167]. For instance, activity‐ and affinity‐based profiling campaigns make use of proximity‐driven electrophiles to identify druggable residues in proteins of interest [168]. Having undergone considerable fine‐tuning of their reactivity, we propose that these reagents have untapped potential for cyclizing randomized peptide libraries on bacteriophages.

For example, sulfur–fluoride exchange (**SuFEx**) chemistry, which employs sulfonyl fluoride or fluorosulfate groups, forms stable S─N, S─O, or S─C bonds with diverse nucleophilic residues (Figure 10I) [169]. While not inherently residue‐specific, **SuFEx** reactivity can be modulated through the substituent (R–SO_2_F or R–OSO_2_F), pH, and the local environment [170, 171]. A related platform, sulfur–triazole exchange (**SuTEx**) chemistry offers additional selectivity via substitution patterns on the triazole ring (Figure 10J) [172, 173]. Compared to **SuFEx**, **SuTEx** reagents exhibit enhanced potency and improved tyrosine selectivity. A recent study by Zanon and coworkers profiled various electrophilic probes across the proteome, confirming that **SuTEx** reagents predominantly target tyrosine residues [174]. Additionally, this study also reaffirmed the long‐recognized selectivity of glyoxal derivatives for arginine side chains [175]. Although lysine reacts more rapidly with glyoxals, only the arginine adduct remains stable, suggesting a viable strategy for proximity‐induced cyclization of arginine‐containing peptides (Figure 10K).

Within chemical proteomics, ligand‐directed (LD) protein labeling has enabled targeted modification of defined protein residues in cells [176]. LD chemistry typically employs weak acylating reagents that undergo proximity‐driven reactions with nucleophilic side chains. Among the electrophiles used in LD chemistry, dibromophenyl benzoate (**BB**) and N‐acyl‐N‐alkyl sulfonamide (**NASA**) are particularly promising for peptide macrocyclization, as they exhibit high selectivity for N‐terminal or ε‐amines of lysine side chains (Figure 10L) [177, 178].

Notably, the electrophiles described in this section are characterized by slow reaction kinetics, making them ideal candidates for designing bifunctional scaffolds. Specifically, when paired with a reactive group for rapid initial modification (e.g., cysteine alkylation), such nonpeptidic scaffolds could enable the selective formation of hybrid MPs on bacteriophages. While **SuTEx**, **SuFEx**, and glyoxals introduce a (small) chemical scar upon cyclization, LD proximity labels, in principle, allow for a *traceless* amide‐bond formation upon macrocyclization.

#### 5.1.5 The Curious Case of N‐Terminal Prolines

In addition to strategies that target amino acid side chains, N‐terminal residues offer another underexplored opportunity for residue‐specific macrocyclization. In addition to those outlined in Section 4.3, N‐terminal proline residues stand out as the presence of a secondary amine confers a distinct reactivity profile [179]. Reactions targeting N‐terminal prolines in proteins include triazene formation with arene diazonium salts [180], oxidative coupling with ortho‐aminophenols or ortho‐catechols [181, 182], and condensation with functionalized ynones [183]. Notably, all of these reactions proceed under mild, aqueous conditions and are compatible with complex biological mixtures.

Given this distinct reactivity, constructing randomized peptide libraries featuring an N‐terminal proline is a promising strategy to access hybrid MPs on phages. However, a major bottleneck lies in the efficient installation of proline residues at the N‐terminus of displayed peptides. Natural filamentous phages, such as fd, readily tolerate most amino acids that directly follow the leader sequence, but the presence of proline at this position inhibits signal peptidase I (SP1)‐mediated processing (Figure 10K) [184, 185]. As periplasmic export of pIII is essential for phage replication, this maturation inhibition severely limits the use of N‐terminal proline in phage display.

To overcome these limitations, we surmise that two potential strategies are worth investigating. First, engineering SP1 variants by directed evolution could enable the processing of leader sequences featuring a proline in the +1 position. Interestingly, the associated growth defect of producing such constructs in *E. coli* could serve as a convenient selection phenotype (Figure 10K) [186, 187]. albeit SP1 being an essential protein for *E. coli* posing a challenge to this strategy. Alternatively, designing peptide libraries with an N‐terminal Met–Pro sequence could prove a more tractable approach (Figure 10J). Following phage assembly, the N‐terminal methionine could be enzymatically removed by methionine aminopeptidase [188], a well‐characterized enzyme that functions *in vitro*.

### 5.2 Pushing the Limits of Phage Display

It might seem intuitive to equate phage compatibility with biocompatibility, but this assumption is unnecessarily conservative. Indeed, bacteriophages are remarkably chemically robust. For example, the infectivity of M13 remains unaffected after exposure to pH 3–11 for 20 min, heating to 70°C for 1 h, or incubation with up to 50% organic co‐solvents such as acetonitrile [50, 189, 190]. Ironically, while these properties frustrate molecular biologists dealing with phage contamination in the lab, from a chemist's perspective, the durability of phages could be leveraged to explore macrocyclization chemistries that extend beyond the typical boundaries of biocompatibility. In this section, we highlight chemical approaches that may be phage‐compatible. Given the vast and rapidly expanding literature on protein modifications, we do not aim to provide an exhaustive list of plausible reactions. Instead, we introduce promising concepts and provide a few illustrative examples of reactions that fall within this category.

#### 5.2.1 Harnessing Slow and Nonselective Reactions

Traditional bioconjugation strategies emphasize rapid reaction kinetics and high selectivity. However, for the formation of hybrid MPs on bacteriophages, these stringent demands can be relaxed. The spatial proximity that can be achieved through an initial cysteine alkylation is able to bring reactive groups in proximity to accelerate otherwise slow and unselective transformations. Moreover, the high chemical resilience of filamentous bacteriophages permits reactions under conditions (e.g., elevated temperatures or nonneutral pH) that would denature most proteins.

One underexplored target in this context is histidine, which reacts with epoxides at pH ∼10 and >65°C (Figure 11A) [191]. While such harsh conditions are generally incompatible with most proteins, they are partially tolerated by bacteriophages. Moreover, Adusumalli and coworkers have recently demonstrated that histidine–epoxide reactions can proceed under milder conditions when proximity effects are exploited [192]. In their study, bifunctional epoxide reagents featuring an aldehyde reversibly condensed with lysine side chains, allowing for the subsequent irreversible labeling of nearby histidine side chains. While overall modification efficiencies remained modest (<50%), combining elevated temperatures with controlled proximity effects (cysteine alkylation) on phages could unlock the potential of epoxides as reagents for selective histidine modification and macrocyclization (Figure 11A).

**FIGURE 11:**
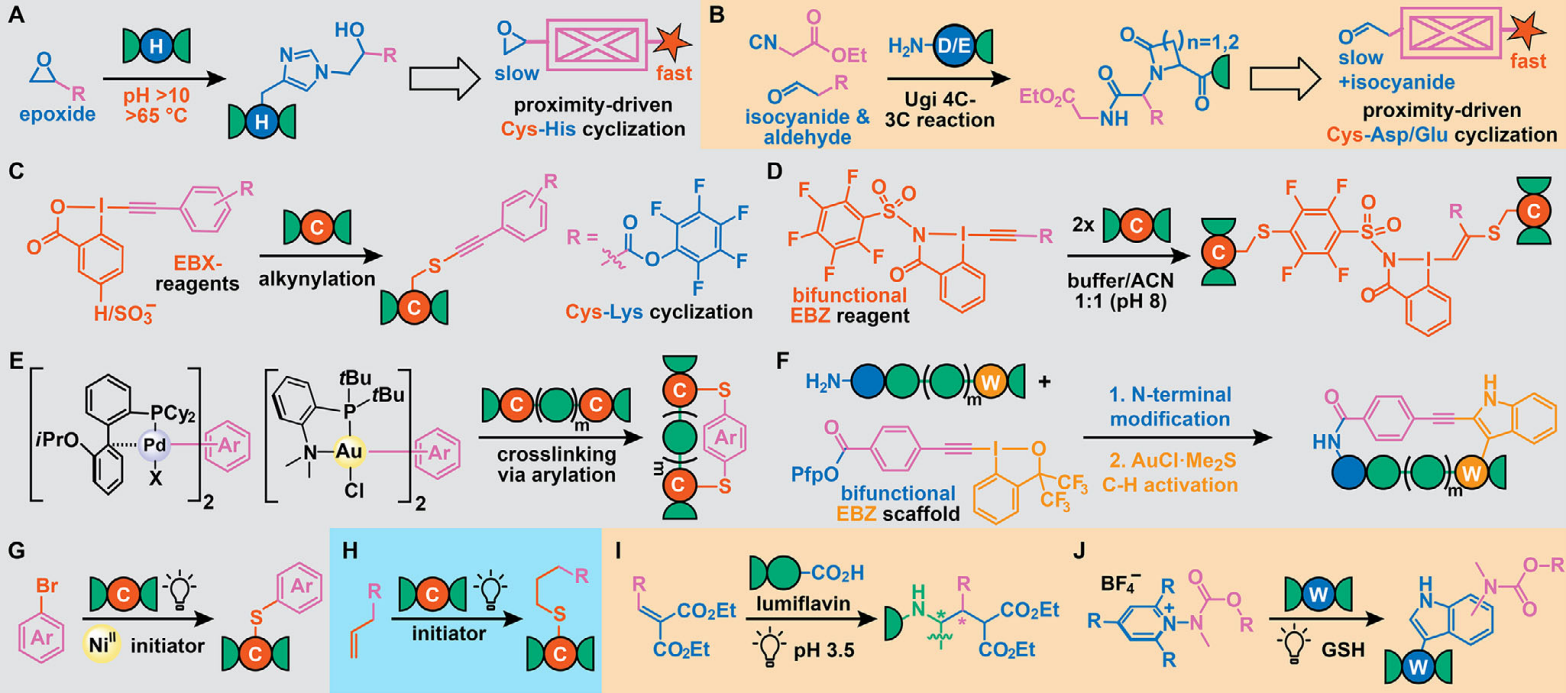
Residue‐specific modification reactions pushing the limits of phage compatibility. (A): Fast cysteine alkylation chemistry can be combined with a slow histidine targeting epoxide reactive handle. A proximity‐driven reaction avoids the usual harsher reaction conditions of epoxide modification. (B): Schematic representation of the 4‐component, 3‐center Ugi multicomponent reaction for selective targeting of N‐terminal aspartate/glutamate residues. (C,D): Structures of hypervalent iodine reagents with improved water solubility which selectively target cysteine residues for Cys‐Lys (C) and Cys‐Cys crosslinking (D). (E): Structures of organometallic palladium‐ and gold‐ based reagents for crosslinking cysteine residues via arylation. (F): Structure of an EBX‐PFP reagent for gold(I)‐catalyzed selective modification of the tryptophan indole side chain following initial modification of the N‐terminus. (G): Photocatalytic arylation of cysteine residues using a nickel(II) initiator. H: Photocatalytic thiol‐ene reactions were applied to produce macrocylic peptides on phages via selective modification of cysteine residues. (I): Structure of a reagent used for selective photoredox‐catalyzed modification of the peptide/protein C‐terminus. (J): Structure of an N‐carbamoylpyridinium‐based reagent for selective modification of the tryptophan side chain.

Multicomponent reactions (MCRs) represent another class of transformations worth re‐evaluating in a bacteriophage display context [193]. In particular, a 4‐component, 3‐center Ugi reaction has been adapted for protein modification by leveraging N‐terminal aspartic or glutamic acid residues as bifunctional handles [194]. These residues can condense with an aldehyde and an isocyanide to form modified 4‐ or 5‐membered lactams (Figure 11B). While the method required long reaction times and high reagent concentrations, phage display offers unique opportunities for improving efficiency. Specifically, aspartate or glutamate residues are readily installed via signal peptidase‐tolerant N‐terminal sequences, and aldehyde‐containing groups can be introduced in close proximity through cysteine alkylation (cf. Section 4.3). In this scenario, only an isocyanide reagent needs to be added in bulk (Figure 11B), potentially overcoming the kinetic and stoichiometric barriers that have limited broader application of Ugi‐based macrocyclization.

#### 5.2.2 Potent but Poorly Soluble (Organometallic) Reagents

With their high tolerance for organic co‐solvents, bacteriophages may also enable the use of peptide modification reagents that exhibit poor water solubility. For example, the Waser lab has developed a suite of ethynylbenziodoxolone(**EBX**) reagents for the rapid and selective alkynylation of cysteine residues (Figure 11C) [195]. While the high selectivity and efficiency of these hypervalent iodine species under dilute conditions make them promising candidates for generating hybrid MPs, their limited water solubility poses a significant challenge.

Case in point, a bifunctional **EBX** scaffold featuring an additional pentafluorophenyl (PFP) ester enabled the programmed modification of a cysteine and a lysine residue, but reactions needed to be performed in pure DMF [196]. To address this challenge, Mishra and coworkers first demonstrated that sulfonylated **EBX** reagents could be employed for the lipophilization of peptides in water (Figure 11C) [197]. More recently, Koutsopetras et al. developed a bifunctional ethynylbenziodazolone (**EBZ**) scaffold which could be employed for cysteine crosslinking in a 1:1 mixture of phosphate buffer and acetonitrile (Figure 11D) [198]. Given that these conditions are likely compatible with bacteriophages, it is plausible that **EBX**‐ and **EBZ**‐based bifunctional scaffolds could soon be applied to the selection of hybrid cyclic peptides.

Additionally, organometallic species have also become popular for modifying or cyclizing unprotected linear peptides in water‐co‐solvent mixtures. Among the available approaches, palladium‐ and gold‐based reagents have been employed for S‐arylation chemistry to staple cysteine‐ (and/or lysine‐) containing peptides and proteins to yield stable S‐aryl conjugates (Figure 11E) [199, 200, 201, 202, 203, 204, 205]. Compared to the arylation by S_N_Ar reagents (cf. Section 3.2), transition metal‐based arylation reagents have a wider substrate scope and can alkylate cysteines with electron‐neutral or electron‐rich aryls.

Although reaction rates are typically slow and require stoichiometric amounts of noble metal species, Liu and coworkers have recently employed Au(I) species in concert with **EBX** reagents for the catalytic C‐H activation of the indole side‐chain in tryptophan (Figure 11F) [206]. In this approach, the hypervalent iodine species was first incorporated by selectively alkylating an N‐terminal amine (or lysine residue) with an **EBX**‐**PFP** ester. In the next step, the modified peptide underwent smooth and catalytic C‐H activation with a nearby indole side‐chain in presence of various Au(I) complexes (e.g. AuCl·Me_2_S) to yield the desired MPs. However, further optimization of reaction conditions is needed for the future application to phage display protocols, as cyclization reactions were carried out in neat organic solvents.

#### 5.2.3 Photocatalytic Modification Reactions

Lastly, visible light‐mediated transformations are inherently biocompatible, as photochemical reactions typically proceed efficiently in aqueous media at room temperature. Visible light, a soft and traceless “reagent,” is also more compatible with labile biomacromolecules than UV light, making it an ideal tool for peptide and protein modifications [207, 208, 209]. Once again, cysteines have featured prominently in the suite of developed transformations, as thiols can undergo smooth arylation or thiol‐ene reactions (Figure 11G,H) [159, 210, 211]. Notably, Ming and coworkers have recently demonstrated that the latter is readily compatible with standard phage display cycles. Specifically, employing 1,3,5‐triallyl‐1,3,5‐triazinane‐2,4,6‐trione (**TAIC**), a three‐fold symmetric crosslinker, and lithium phenyl‐2,4,6‐trimethylbenzoylphosphinate as initiator enabled the authors to select bicyclic peptides with nanomolar affinity against CypA [212].

While further, general photochemical modification of peptides and proteins has been extensively reviewed elsewhere [207, 208, 209], we would like to briefly highlight chemoselective transformations that unlock reactivity at residues rarely targeted for bioconjugation. For example, Bloom and coworkers demonstrated that photoredox catalysis using a flavin photocatalyst enables selective decarboxylative alkylation at the C‐terminus of peptides and proteins (Figure 11I) [213]. This selectivity arises from the distinct oxidation potential of the C‐terminal carboxylate group compared to internal aspartate and glutamate residues. The reaction proved most efficient in cesium carbonate buffer at pH 3.5, acidic conditions that should be readily compatible with bacteriophages.

More recently, Tower and colleagues reported a promising example of a chemoselective conjugation of tryptophan residues by employing N‐carbamoylpyridinium salts (Figure 11J) [214]. The reaction is proposed to proceed via photoinduced electron transfer (PET) between tryptophan and the pyridinium salt, leading to N─N bond fragmentation and subsequent formation of an N─C bond between the carbamoyl group and the C2 position of tryptophan. In the presence of 1 mM glutathione (GSH), the method achieved high conversions (>85%) for selective modification of peptides (∼1–3 kDa). Notably, a red‐shifted pyridinium salt enabled effective labeling (>90% conversion) of photolabile lysozyme within 45 min. Given that phages have been shown to tolerate irradiation and competing tryptophan residues are rare on phage coat proteins, these results augur favorably for applying this photoconjugation strategy in future phage display selections of natural product‐like MPs.

In light of the potential cross‐reactivity of other residues on bacteriophages, it is important to stress that the activation by light provides an additional layer of spatiotemporal control. Specifically, for the preparation of diverse MP libraries on phages, photolabile species could be selectively activated only after their installation in close proximity to their desired reaction partner. This approach would enable short reaction times while minimizing unwanted side reactions with competing functional groups present in phage‐coat proteins.

## 6 Summary and Outlook

### 6.1 What Makes a Cyclization Strategy Ideal for the Selection of Hybrid MPs?

In this Review, we have presented a detailed overview of established and emerging strategies for peptide diversification and macrocyclization that are (potentially) compatible with phage display. Collectively, these approaches highlight several key features that a successful strategy for the generation of natural‐product‐like hybrid MPs on phages should possess.

First, at least one of the employed modification reactions must satisfy stringent criteria with respect to both selectivity and reactivity. Minimizing cross‐reactivity with residues on phage coat proteins is essential, not only to avoid the formation of side products that complicate downstream deconvolution of binders, but also to preserve phage infectivity by preventing hypermodification or undesired crosslinking of phage particles. At the same time, the modification chemistry must proceed rapidly and in a largely irreversible manner to enable efficient macrocyclization under the highly dilute conditions typical of phage display (<0.1 nM) and to ensure stability of the resulting constructs over extended timescales (days). Importantly, modified phages must also remain compatible with iterative amplification and selection cycles, placing additional constraints on scaffold stability and the extent of phage coat modification. From a practical standpoint, cyclization strategies should furthermore operate in a one‐pot fashion with minimal user intervention following reagent addition. With these constraints in mind, it is unsurprising that early approaches predominantly relied on symmetrical cysteine‐reactive crosslinkers (cf. Section 3), whereas more recent efforts have explored enzymatic ligation strategies to achieve selective peptide cyclization.

The incorporation of asymmetric scaffolds to access natural‐product‐like hybrid MPs imposes an additional level of complexity, as it requires the programmed modification of two orthogonal functional groups. Importantly, the use of a fast and selective first transformation (e.g. cysteine modification) can substantially relax the requirements for the second cyclization step. Once a scaffold is tethered in close proximity to a second reactive residue, otherwise slow or poorly selective reactions can become viable through proximity‐driven effects. To date, N‐terminal modification strategies have proven especially powerful in this regard, either by exploiting the unique reactivity of 1,2‐aminothiols in N‐terminal cysteines or the enhanced nucleophilicity of N‐terminal α‐amines at near‐neutral pH. As highlighted throughout this Review, leveraging proximity effects to harness otherwise slow or insufficiently selective reactions may dramatically expand the chemical space accessible to phage‐display selections of hybrid MPs. This prospect is particularly compelling given the exceptional robustness of bacteriophages, which tolerate temperatures, pH ranges, and solvent conditions that extend well beyond the boundaries of traditional biocompatibility.

Nevertheless, several less obvious design considerations must also be taken into account when developing cyclization chemistries for phage display. First, the employed reactivity should not excessively constrain the amino acid diversity of randomized peptide libraries. Broadly reactive electrophiles, for instance, risk introducing multiple competing reaction partners within a single peptide sequence, thereby complicating both the structural assignment and resynthesis of selected binders. Second, cyclization reactions should minimize the introduction of large chemical “scars” wherever possible, as bulky or pharmacologically undesirable motifs may negatively affect the physicochemical properties of selected MPs and complicate subsequent medicinal chemistry optimization. In this regard, cyclization scaffolds should remain amenable to derivatization and compatible with SAR optimization. Finally, asymmetric scaffolds intended for peptide diversification should remain synthetically accessible and chemically stable, such that they can be prepared, handled, and stored without requiring specialized expertise in synthetic organic chemistry. Beyond synthetic accessibility and selectivity, future cyclization strategies should also provide access to broad conformational diversity, as only a limited subset of macrocyclic topologies may be poised to generate high‐affinity binders against challenging targets.

Combined, the successful development of hybrid MP selection strategies requires balancing chemical reactivity, residue selectivity, scaffold accessibility, and preservation of library diversity. The continued convergence of advances in bioconjugation chemistry, enzymatic engineering, and peptide design is therefore likely to further expand the scope of architectures accessible by phage display, progressively closing the gap between genetically encoded peptide libraries and the structural sophistication of hybrid MPs found in Nature.

### 6.2 Toward a Unified Framework for the Design, Selection, and Optimization of Hybrid MPs

Over the past decades, bacteriophage display has evolved from a platform for the identification of linear peptide binders into a versatile discovery engine capable of accessing increasingly complex macrocyclic and natural‐product‐like architectures. This transformation has been driven in large part by advances in peptide cyclization chemistry and residue‐selective modification strategies, which have progressively expanded the structural space accessible to genetically encoded libraries. However, despite these developments, the exploration of hybrid MPs remains largely opportunistic rather than systematic, with chemical space being expanded on a case‐by‐case basis rather than in a coordinated fashion. Looking forward, a central opportunity lies in moving from such isolated expansions toward a more deliberate charting of both the chemical and functional space accessible to phage‐displayed hybrid MPs. In this context, future platforms will not only aim to generate binders, but to map and navigate a multidimensional landscape defined by structure, reactivity, and developability, ultimately enabling more predictive and controlled molecular discovery.

Among the most direct routes to further expand this chemically‐enabled design space is the incorporation of additional building blocks directly at the genetic level. In particular, noncanonical amino acids (ncAAs) provide a powerful means to encode orthogonal reactivity and structural diversity into peptide libraries prior to chemical diversification or selection [215, 216, 217, 218]. ncAAs can introduce functional groups that are absent in the repertoire of canonical amino acid, thereby enabling selective post‐translational modification, cyclization, or late‐stage diversification under mild conditions. Importantly, ncAAs also create a more direct link between genetic information and synthetic chemical space, effectively extending the sequence‐encoded alphabet available for molecular design. Despite this promise, their broader application in hybrid MP selection by phage display remains limited by challenges in translational incorporation efficiency and the context dependency of expression systems.

In parallel, computational approaches, particularly machine learning (ML), are beginning to offer complementary opportunities for navigating the expanding chemical space of hybrid MPs. While ML‐guided peptide and MP design has advanced rapidly in recent years [219, 220, 221]. models capable of reliably handling hybrid molecules that combine peptide backbones with nonpeptidic scaffolds remain in their infancy. A central challenge lies in the absence of established molecular representations and descriptors that adequately capture the interplay between peptide sequence, macrocyclic constraint, and synthetic scaffolds [222, 223]. Standard protein or small‐molecule feature sets could be insufficient to describe these hybrid systems in a unified manner, complicating both model training and generalization. Moreover, the scarcity of high‐quality, systematically generated datasets linking hybrid MP sequences to binding, structure, and functional outcomes further limits predictive performance.

Importantly, phage display already provides access to experimentally selectable functional readouts beyond affinity alone, including proteolytic stability through selections in protease‐rich environments [224], as well as functional binding under increasingly complex biological contexts, among which selections performed in the presence of live cells [225]. These selection modalities provide a unique opportunity to generate datasets that directly encode developability and functional performance under biologically relevant conditions. Future iterations of ML models are therefore unlikely to be restricted to affinity prediction alone. Instead, they could be expanded to incorporate experimentally relevant pharmacological and developability parameters, including proteolytic stability, permeability, solubility, aggregation propensity, and functional performance in cellular contexts, thereby enabling multi‐parameter optimization directly within the design loop. Realizing this vision will require not only improved molecular representations, but also dedicated experimental campaigns designed to generate structurally diverse and mechanistically annotated datasets that jointly capture binding behavior and key pharmacological properties of hybrid libraries.

Overall, the convergence of expanded chemical toolsets, ncAA incorporation strategies, and data‐driven computational design is poised to reshape the future landscape of hybrid MP discovery. Rather than relying on incremental chemical modification of existing scaffolds, future phage display platforms are likely to enable an integrated, multi‐objective design–selection loop in which binding affinity, pharmacological developability, and selection compatibility are considered simultaneously from the outset. In this envisioned framework, chemical diversification, genetic encoding, and machine learning‐guided prioritization operate in concert, allowing both the generation and ranking of hybrid macrocycles within a unified experimental–computational workflow. Such an approach would move the field beyond *post hoc* optimization toward the prospective design of ligands with predefined functional profiles, including stability, solubility, and protease resistance alongside target engagement. Ultimately, this integration has the potential not only to expand the accessible chemical space of peptide‐based ligands, but also to substantially accelerate early‐stage drug discovery by reducing reliance on iterative downstream optimization cycles.

## Conflicts of Interest

The authors declare no conflicts of interest.

## Data Availability

Data sharing not applicable to this article as no datasets were generated or analyzed during the current study.
